# Relational *vs* representational social cognitive processing: a coordinate-based meta-analysis of neuroimaging data

**DOI:** 10.1093/scan/nsad003

**Published:** 2023-01-25

**Authors:** Maria Arioli, Zaira Cattaneo, Simone Parimbelli, Nicola Canessa

**Affiliations:** Department of Human and Social Sciences, University of Bergamo, Bergamo 24100, Italy; Department of Human and Social Sciences, University of Bergamo, Bergamo 24100, Italy; IRCCS Mondino Foundation, Pavia 27100, Italy; IUSS Cognitive Neuroscience (ICoN) Center, Scuola Universitaria Superiore IUSS, Pavia 27100, Italy; IUSS Cognitive Neuroscience (ICoN) Center, Scuola Universitaria Superiore IUSS, Pavia 27100, Italy; Istituti Clinici Scientifici Maugeri IRCCS, Cognitive Neuroscience Laboratory of Pavia Institute, Pavia 27100, Italy

**Keywords:** relational, representational, mentalizing, action observation, temporo-parietal junction

## Abstract

The neurocognitive bases of social cognition have been framed in terms of representing others’ actions through the mirror system and their mental states via the mentalizing network. Alongside representing another person’s actions or mental states, however, social cognitive processing is also shaped by their (mis)match with one’s own corresponding states. Here, we addressed the distinction between representing others’ states through the action observation or mentalizing networks (i.e. representational processing) and detecting the extent to which such states align with one’s own ones (i.e. relational processing, mediated by social conflict). We took a meta-analytic approach to unveil the neural bases of both relational and representational processing by focusing on previously reported brain activations from functional magnetic resonance imaging studies using false-belief and action observation tasks. Our findings suggest that relational processing for belief and action states involves, respectively, the left and right temporo-parietal junction, likely contributing to self-other differentiation. Moreover, distinct sectors of the posterior fronto-medial cortex support social conflict processing for belief and action, possibly through the inhibition of conflictual representations. These data might pave the way for further studies addressing social conflict as an important component of normal and pathological processing, and inform the design of rehabilitative treatments for social deficits.

## Introduction

A core topic in social cognitive neuroscience concerns the role of the mirror and mentalizing brain networks (Arioli *et al.*, 2018a; Geiger *et al.*, 2019; Van Overwalle and Baetens, 2009) in understanding others’ behaviours * * and decoding their intentions and feelings (Arioli *et al.*, 2018b, 2021a; Heleven *et al.*, 2018; Van Overwalle, 2009). Notably, these networks are generally considered to underpin a neural basis for representing these different components of social understanding.

The mirror network includes inferior frontal, premotor and inferior parietal regions which are activated both when performing an action and when observing the same action performed by someone else (Urgesi *et al.*, 2006; Cattaneo and Rizzolatti, 2009; Avenanti and Urgesi, 2011; Campbell and Cunnington, 2017; Jeon and Lee, 2018). This direct matching between action execution and observation is considered to underpin a variety of social functions mediated by a mental representation of another’s actions , such as recognition (Bonini, 2017), imitation learning (Buccino *et al.*, 2005; Oztop *et al.*, 2013) and the decoding of intentions signalled by visuomotor and/or contextual cues (Iacoboni *et al.*, 2005; Arioli and Canessa, 2019). The mentalizing system, including the medial prefrontal cortex (mPFC), the temporo-parietal junction (TPJ), the medial precuneus/posterior cingulate cortex and the temporal poles (Schurz *et al.*, 2020; Arioli *et al.*, 2021a), is rather activated when—in the lack of informative visuomotor cues—others’ intentions must be inferred in terms of mental states such as thoughts and beliefs (Molenberghs *et al.*, 2016). Importantly, the use of different terms such as ‘theory of mind’, ‘mentalizing’, ‘mind-reading’ or ‘perspective-taking’—typically associated with partly overlapping and partly different meanings—has generated some confusion in the related literature (Schaafsma *et al.*, 2015; Schurz *et al.*, 2020). We therefore chose to use only the term ‘mentalizing’, that has been associated primarily with the results of neuroimaging studies aimed at characterizing the brain regions involved in representing and understanding others’ mental states (i.e. a ‘mentalizing’ network, e.g. Hoskinson *et al.*, 2019).

Along with representing others’ intentional states, however, evaluating whether they (mis)match with our own ones might represent another crucial prerequisite for effective social communication and interactions. This distinction has been recently conceptualized between representing others’ intentions (be it in terms of mental states or actions), i.e. representational processing, and detecting the extent to which such representation is (mis)matching with our own state, i.e. relational processing based on monitoring a ‘social conflict’, i.e. a conflict/mismatch between self- and other-related actions or states (Deschrijver and Palmer, 2020).

Well before this proposal, however, both representational and relational processing had been implemented in neuroimaging studies with either actions or mental states (e.g., beliefs) as ‘target’ stimuli.

In the former case, studies addressing action representation typically compared conditions depicting another’s action *vs* no visible human movement (e.g. scrambled images or object mechanical movements), without action execution (Zhang *et al.*, 2017; Morales *et al.*, 2019). In contrast, studies of action conflict monitoring contrasted conditions eliciting misalignment between performed and attended actions (socially incongruent condition) with conditions in which a same action is both executed and observed (socially congruent condition) (Cracco *et al.*, 2018; Darda *et al.*, 2018; Darda and Ramsey, 2019).

In the case of mentalizing, most neuroimaging studies used false-belief tasks requiring to make inferences on another’s mental states *vs* non-mental (e.g. physical) events (Alderson-Day *et al.*, 2016; Wysocka *et al.*, 2020). Notably, the ‘false-belief’ and ‘true-belief’ conditions of this task implicitly elicit, respectively, mismatching (socially incongruent condition) and matching (socially congruent condition) representations across the subject and the story character. While comparing these conditions should thus unveil the regions involved in belief conflict monitoring (Sommer *et al.*, 2018; Cracco *et al.*, 2020), this contrast is rather commonly interpreted in terms of mental representation (e.g. Özdem *et al.*, 2019). In ‘false-belief’ conditions, the mismatch between one’s own and the other’s knowledge is supposed to prompt the construction of her/his model of the world, i.e. mentalizing (Phillips and Norby, 2019), rather than supporting the processing of social conflict. Interpreting results under a relational (i.e. social conflict), rather than representational, framework might thus help characterizing mechanisms of social cognition that are shared across different social domains, such as action perception and belief understanding, as well as during moral decision-making and understanding of irony, lies and humour (Deschrijver and Palmer, 2020). For example, the appreciation of irony and sarcasm may emerge from the interplay between the social verbal cues given by others and the world as interpreted by oneself, rather than reflecting other’s mental-state representation *per se* (Deschrijver and Palmer, 2020).

On this basis, it has been suggested that assessing the correspondence between another’s and our own model of the world might represent the crucial process, providing the most critical information, for social understanding (Deschrijver and Palmer, 2020). The latter, and more generally human communication, may depend more on assessing how well one’s knowledge aligns with others’ knowledge than on inferring their mental states. A failure in processing the difference between one’s own and others’ lines of thought might thus be expected to result in social impairments. An ideal benchmark for this hypothesis is represented by autism spectrum disorder, in which social deficits might reflect altered mechanisms of social conflict monitoring rather than difficulties in representing others’ minds, i.e. ‘mindblindness’ (e.g. Nijhof *et al.*, 2018). This view fits with the observation of TPJ—in which altered activity has been previously reported in autism (Donaldson *et al.*, 2018; Yuk *et al.*, 2018)—as the common neural basis of mechanisms for monitoring social conflicts conveyed both by action perception (Marsh *et al.*, 2016) and belief understanding (Bardi *et al.*, 2017). Moreover, neuromodulation of TPJ activity has been shown to influence social conflict monitoring (Sowden and Shah, 2014; Nobusako *et al.*, 2017), but not representational measures of actions or mental states (Hogeveen *et al.*, 2015; Santiesteban *et al.*, 2015).

Some studies, however, failed to report a specific TPJ involvement in social conflict monitoring when comparing false and true-belief conditions (Abraham *et al.*, 2010; Schneider *et al.*, 2014) or socially incongruent and congruent conditions in the action–perception domain (Campbell *et al.*, 2018). Moreover, increasing evidence suggests that social conflict monitoring involves regions other than the TPJ, such as the insula for action perception (Crescentini *et al.*, 2011; Cross *et al.*, 2013) and the inferior parietal lobule (IPL) for both action observation (Wang *et al.*, 2011) and false belief (Rothmayr *et al.*, 2011). Therefore, the putative exclusive role of TPJ in social conflict monitoring (Marsh *et al.*, 2016; Bardi *et al.*, 2017) needs further supporting evidence. A related gap concerns the proposal of TPJ as a ‘relational’ hub common to distinct social–cognitive domains, possibly receiving input from the motor cortex *vs* areas underlying mental representations when monitoring action conflict *vs* belief conflict, respectively (Deschrijver and Palmer, 2020).

On this basis, we performed a coordinate-based meta-analysis of previous functional magnetic resonance imaging (fMRI) studies with false-belief and action observation tasks to investigate the specific and/or overlapping neural bases of (i) representational processing and (ii) relational processing based on social conflict monitoring. Available evidence (e.g. Campbell *et al.*, 2018) suggests an involvement of TPJ both for belief and action conflict monitoring. According to Deschrijver and Palmer’s (2020) hypothesis, a prominent engagement of TPJ in relational, instead of representational, processing would support a reframing of mentalizing (and probably also other social–cognitive domains) in terms of relational rather than representational processing.

## Materials and methods

### Rationale of the meta-analytic approach

We aimed to identify the brain regions consistently associated with relational processing (i.e. social conflict monitoring), over and beyond its requirements in terms of representational processing (Deschrijver and Palmer, 2020), by focusing on false-belief (Sommer *et al.*, 2018) and action observation (Biagi *et al.*, 2016) tasks, respectively. We first pursued this goal with activation likelihood estimation (ALE), a coordinate-based meta-analytic approach using coordinates of peak locations to summarize and integrate published findings (Turkeltaub *et al.*, 2002). This approach allows to overcome the typical limitations inherent in single neuroimaging experiments, e.g. sensitivity to experimental and/or analytic procedures, lack of replication studies and small sample sizes (Carp, 2012). These constraints are known to increase the likelihood of false negatives (Button *et al.*, 2013), thus pushing researchers towards procedures that, conversely, might promote false positives (Eklund *et al.*, 2016; Müller *et al.*, 2018).

We first ran four separate ALE analyses addressing the neural processing of belief relational, belief representation, action relational and action representation processes in healthy individuals. Subsequent conjunction and contrast analyses unveiled both common and specific activations across (i) belief relational and belief representation, (ii) action relational and action representation and (iii) belief relational and action relational processing. We did not perform comparison/conjunction analyses of belief and action representational processing because they would not fit our purpose of clarifying the neural bases of relational (*vs* representational) processing.

All the inclusion criteria for each dataset were selected by the first author and then checked and approved by the other authors. This procedure, entailing a double check by independent investigators, was aimed to reduce the chances of a selection bias (Müller *et al.*, 2018). The selection process began in September 2020. M.A and S.P. independently screened the papers for the meta-analyses on false belief and action observation, respectively, and weekly meetings were scheduled to resolve doubts. To further improve the quality of the selection process, at the end of this first selection stage, the two authors exchanged their databases for a cross-check. Finally, the two databases were also checked and approved by the other two authors (Z.C. and N.C.).

### Literature search and study selection

#### Neural bases of belief representation and belief relational processing.

**Fig. 1.**
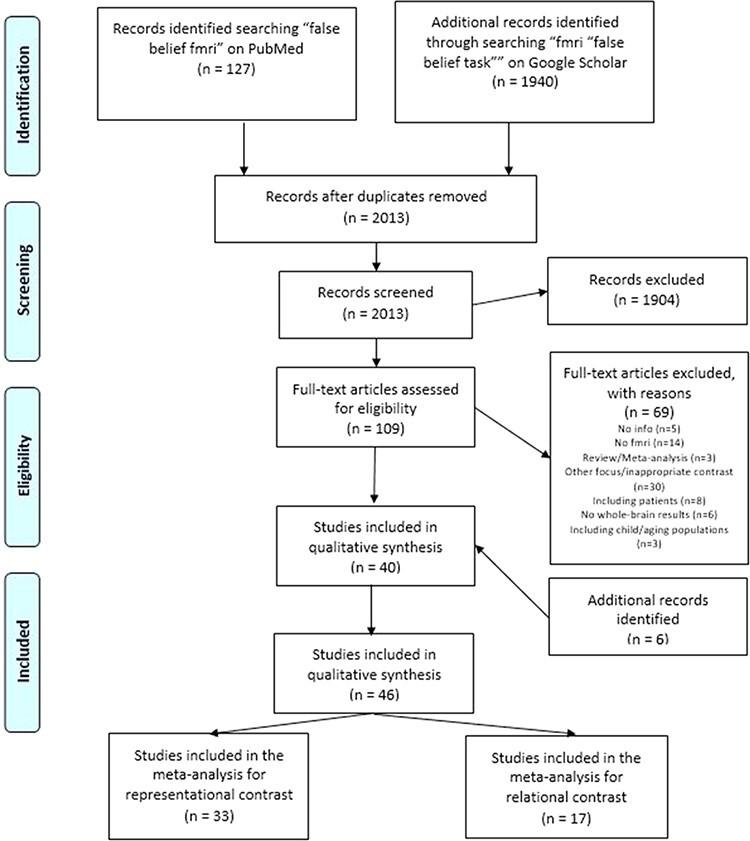
PRISMA flowchart of literature search and selection process for false-belief task

Following Preferred Reporting Items for Systematic Reviews and Meta-Analysis (PRISMA) guidelines (Liberati *et al.*, 2009; Page *et al.*, 2021) and the guidelines for neuroimaging meta-analysis (Müller *et al.*, 2018), we started our survey of the relevant literature by searching for ‘false-belief fMRI’ on PubMed (https://www.ncbi.nlm.nih.gov/pubmed/; research date: 24 August 2021) and by constraining this search to studies on human subjects and written in English. Additional records were identified by searching for ‘fMRI “false-belief task”’ on Google Scholar (https://scholar.google.com/; research date: 24 August 2021). In the latter search, we used quotation marks to retrieve only papers reporting the ‘false-belief task’ in the text. While the search was limited to papers written in English, we did not apply temporal filters (e.g. specific years of publication). After duplicate removal, a preliminary pool of 2013 studies was first screened by titles and then by abstracts. We retained only those studies fulfilling the following selection criteria (see Figure 1 for details on the procedure for study selection):

- Studies written in the English language.
- Empirical fMRI studies, while excluding review and meta-analysis studies and those employing other techniques, to ensure comparable spatial and temporal resolution.
- Studies reporting whole-brain activation coordinates, rather than regions of interest (ROIs) or results of small-volume correction (SVC). Studies based on ROIs or SVC must be excluded because a prerequisite for fMRI meta-analyses is that convergence across experiments is tested against a null hypothesis of random spatial associations across the entire brain under the assumption that each voxel has the same a priori chance of being activated (Eickhoff *et al.*, 2012; Müller *et al.*, 2018).
- Studies including drug-free and non-clinical participants to prevent possible differences in brain activity associated with pharmacological manipulations or neuro-psychiatric diseases other than those under investigation.
- Studies with adult subjects (age range: 18–60 years).
- A minimum of five participants included in the final analyses, as usually advised for neuroimaging meta-analyses (e.g. Zhang *et al.*, 2019).
- Studies using the false-belief task to investigate the neural bases of making inferences on another’s beliefs. We selected only studies performing either of these contrasts or both:
  - Inferences on ‘true belief > inferences on physical or perceptual aspects’ and inferences on ‘false belief > inferences on physical or perceptual aspect’. While the first contrast allows to isolate belief representation, the latter contrast leads to the inclusion of studies using ‘false belief’ as a target condition that entails not only representing another mind but also representing the self-other distinction and social conflict. Importantly, most of the published studies with ‘false belief’ as a target condition used the ‘false photograph’ as a control condition in which subjects are required to represent the outdated content of a physical representation such as a photograph. The rationale for choosing this control is that it makes the target and control conditions structurally equivalent (including for the presence of conflict) and differing for their requirements in terms of processing mental states. Importantly, however, there is no evidence that this condition with perceptual conflict can control for social conflict.
  - This selection, however, retained studies contrasting belief and non-belief conditions, while excluding studies in which beliefs were contrasted with low-level baseline conditions such as rest or visual fixation.
  - Studies using these contrasts to unveil the neural bases of representing another’s mental states were included in the ‘belief representational’ meta-analysis.
  - Inferences on another’s false-belief (socially incongruent/mismatching condition, in which the other person’s mental representation of the situation differs from the participant’s own belief) > inferences on another’s true-belief (socially congruent condition, in which the participant’s and another’s beliefs match with each other). Studies using this contrast to isolate the regions engaged in processing the social conflict that is present only in the false-belief condition were included in the ‘belief relational’ meta-analysis.

Studies not reporting some of the required information (e.g. participants’ number or age, or coordinates for contrasts of interest) were excluded. Starting from an initial screening of 2013 titles and abstracts, 109 papers deemed as potentially relevant were fully reviewed based on the aforementioned selection criteria (Figure 1). We thus excluded: 3 review/meta-analysis articles; 6 studies using ROIs or SVC; 3 studies focused on children or ageing populations; 30 studies focusing on other topics or using inappropriate contrasts; 8 studies focused on clinical populations; 14 studies employing techniques other than fMRI and 5 studies not reporting all the required information. This selection phase resulted in a final set of 40 studies fulfilling our selection criteria.

We then expanded our search for other potentially relevant studies by carefully examining both the studies quoting, and those quoted by, each of these papers, alongside previously published review and meta-analysis papers on similar topics (Arioli and Canessa, 2019; Deschrijver and Palmer, 2020). This second phase highlighted six further studies fitting our search criteria. Studies were classified as ‘relational mentalizing’ if they required participants to infer a belief conflict and ‘representational mentalizing’ if they involved belief understanding. Overall, this procedure led to include in the ALE ‘belief representational’ meta-analysis 33 previously published studies (Supplementary Table S1), resulting from 34 experiments (individual comparisons reported) with 769 subjects and 405 activation foci. Instead, the ALE ‘belief relational’ meta-analysis included 17 previously published studies (Supplementary Table S2), resulting from 17 experiments (individual comparisons reported) with 323 subjects and 202 activation foci.

#### Neural bases of action representation and action relational processing.

**Fig. 2.**
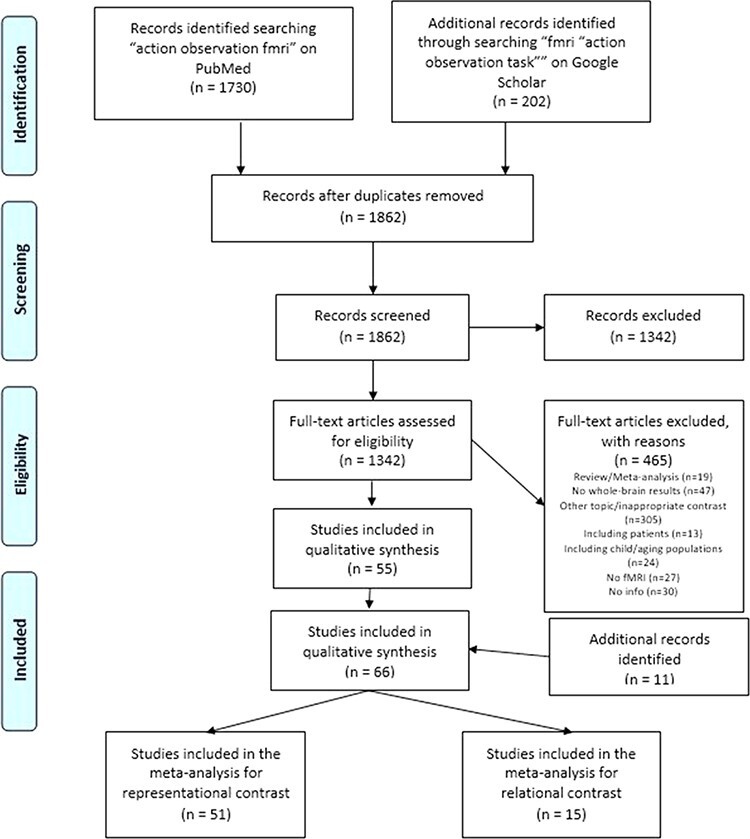
PRISMA flowchart of literature search and selection process for action observation tasks.

We started our survey of the relevant literature by searching for ‘action observation fMRI’ on PubMed (https://www.ncbi.nlm.nih.gov/pubmed/; research date: 24 August 2021) and by constraining this search to studies on human subjects and written in English. Additional records were identified by searching for ‘fMRI “action observation task”’ on Google Scholar (https://scholar.google.com/; research date: 24 August 2021). In the latter search, we used quotation marks to retrieve only papers reporting ‘action observation task’. While the search was limited to papers written in English, we did not apply temporal filters (e.g. specific years of publication). After duplicate removal, a preliminary pool of 1862 studies was first screened by titles and then by abstracts (Figure 2). While the methodological selection criteria were the same as mentioned earlier ((i)–(vi)), here we selected only studies addressing action observation tasks with either of these contrasts or both:

- Action observation > control condition with no visible human action (e.g. objects movement, static pictures of humans or scrambled images), without action execution. This selection retained studies contrasting the visual processing of human actions and complex non-human action stimuli while excluding studies in which actions were contrasted with low-level baseline conditions such as rest or visual fixation. Studies using this contrast to unveil the neural bases of representing observed actions were included in the ‘action representational’ meta-analysis.
- Socially incongruent action (when there is a mismatch between the action performed by the participants and the one that they observe being performed by someone else) > socially congruent action (when the participants’ action is identical to the observed one). Importantly, this comparison is not directly informative about whether the individual represents the other’s action, as the difference between the two conditions refers to a mismatch (i.e. social conflict) between the participant’s and other’s actions, and not to a particular action as such. Studies using this type of contrast were included in the ‘action relational’ meta-analysis.

Starting from an initial screening of 1862 titles and abstracts, 520 papers deemed as potentially relevant were fully reviewed based on the aforementioned selection criteria (Figure 2). We thus excluded: 19 review or meta-analysis articles; 27 studies employing techniques other than fMRI; 47 studies using ROIs or SVC; 24 studies focused on children or ageing populations; 30 studies not reporting all the required information; 305 studies focusing on other topics or using inappropriate contrasts, and 13 studies focused on clinical populations. This selection phase resulted in 55 studies fulfilling our selection criteria.

We then expanded our search for other potentially relevant studies by carefully examining both the studies quoting, and those quoted by, each of these papers, alongside previously published meta-analyses on the neural bases of action observation processing (Cracco *et al.*, 2018; Arioli and Canessa, 2019; Darda and Ramsey, 2019; Deschrijver and Palmer, 2020). This second phase highlighted 11 further studies fitting our search criteria. Studies were classified as ‘relational action processing’ if they required participants to monitor an action conflict and ‘representational action processing’ if they involved action perception. Overall, this procedure led to include in the ALE ‘action representational’ meta-analysis 51 previously published studies (Supplementary Table S3), resulting from 52 experiments (individual comparisons reported) with 933 subjects and 1006 activation foci. Instead, the ALE ‘action relational’ meta-analysis included 15 previously published studies (Supplementary Table S4), resulting from 15 experiments (individual comparisons reported) with 308 subjects and 194 activation foci.

### Activation likelihood estimation

We performed four ALE analyses, using the GingerALE 3.0.2 software (Eickhoff *et al.*, 2009), to identify regions consistently associated with (i) belief representational processing, (ii) belief relational processing, (iii) action representational processing and (iv) action relational processing. We followed the analytic procedure previously described by Arioli *et al.* (2020) and Arioli and Canessa (2019), based on Eickhoff *et al.* (2012). Importantly, the inclusion of multiple contrasts/experiments from the same set of subjects can generate dependence across experiment maps and thus decrease the validity of meta-analytic results. To prevent this issue, for each meta-analysis we adjusted for within-group effects by pooling the coordinates from all the relevant contrasts of a study into one experiment (Turkeltaub *et al.*, 2002). The number of experiments included in most of these meta-analyses is in line with the recent prescriptions for ALE (Eickhoff *et al.*, 2016; Müller *et al.*, 2018), suggesting a minimum of 17 experiments to ensure that results would not be driven by single experiments (Xiong *et al.*, 2019). Only the analysis on action relational processing included less than 17 experiments (i.e. 15). However, this numerosity is in line with van Veluw and Chance (2014) and Xiong *et al.*’s (2019) meta-analyses on social processing in healthy individuals.

In all meta-analyses, activation foci were initially interpreted as the centres of three-dimensional Gaussian probability distributions to capture the spatial uncertainty associated with each individual coordinate. All coordinates were reported in the MNI space or converted into this space using the automatic routine implemented in GingerALE. The three-dimensional probabilities of all activation foci in a given experiment were then combined for each voxel, resulting in a modelled activation (MA) map. The union of these maps produces ALE scores describing the convergence of results at each brain voxel (Turkeltaub *et al.*, 2002). To distinguish ‘true’ convergence across studies from random convergence (i.e. noise), the ALE scores are compared with an empirically defined null distribution (Eickhoff *et al.*, 2012). The latter reflects a random spatial association between experiments, with the within-experiment distribution of foci being treated as a fixed property. A random-effects inference is thus invoked by focusing on the above-chance convergence between different experiments and not on the clustering of foci within a specific experiment. From a computational standpoint, deriving this null hypothesis involved sampling a voxel at random from each MA map and taking the union of the resulting values. The ALE score obtained under this assumption of spatial independence was recorded, and the permutation procedure was iterated 1000 times to obtain a sufficient sample of the ALE null distribution. The ‘true’ ALE scores were tested against the ALE scores obtained under the null distribution and thresholded at *P* < 0.05, corrected for cluster-level family-wise error, and the cluster-level threshold was set at *P* < 0.01 to identify the above-chance convergence in each analysis.

The resulting maps were then fed into direct comparisons and conjunction analyses, within GingerALE, to unveil the common and specific brain activations across (i) belief representation and belief relational, (ii) action representation and action relational, and (iii) belief relational and action relational processing.

For each comparison, a conjunction image was created, using the voxel-wise minimum value of the included ALE images, to display the similarity between datasets (Eickhoff *et al.*, 2011). In the same analysis, two ALE contrast images were created and compared by directly subtracting one input image from the other. To correct for sampling errors, GingerALE creates such data by pooling the foci in each dataset and randomly dividing them into two new groupings equivalent in size to the original datasets. An ALE image is created for each new dataset, then subtracted from the other and compared with the true data. Permutation calculations are then used to compute a voxel-wise *P*-value image indicating where the values of the ‘true data’ fall within the distribution of values in any single voxel. To simplify the interpretation of ALE contrast images, significant ALE subtraction scores were converted to Z scores. For contrast analyses, we used a threshold set at *P* < 0.05, using 10 000 permutations, and a minimum volume size of 100 mm^3^.

Anatomical labelling of all clusters was automatically generated by GingerALE (Eickhoff *et al.*, 2012). Moreover, we used the Statistical Parametric Mapping Anatomy Toolbox (v.2.2c; Eickhoff *et al.*, 2005), as well as the AAL template (as implemented in MRIcron; https://www.nitrc.org/projects/mricron) and Neurosynth (https://www.neurosynth.org/locations/), to double-check these localizations.

### Publication bias

The generalizability of coordinate-based meta-analyses is hampered by the exclusion of studies that are not published, typically due to the lack of statistically significant findings. We took two distinct approaches to assure the robustness of our ALE meta-analytic findings against such publication bias (i.e. the higher likelihood of positive, compared with negative, findings to be reported). Namely, we calculated (i) the relationship between number of participants and number of significant findings reported (e.g. foci detected) (David *et al.*, 2013, 2018; Alegria *et al.*, 2016), and (ii) for each cluster of each meta-analysis, the fail-safe number (FSN) (Acar *et al.*, 2018).

The rationale for the former approach is that a negative correlation between sample size and number of foci is typical of analyses with publication bias, where studies with small samples are published only if their results match a priori hypotheses (David *et al.*, 2013). Against this possible confound, for none of the performed ALE meta-analyses we observed a significant negative correlation between sample size and number of foci [action relational: *r*(13) = 0.504, *P* = 0.055; action representation: *r*(50) = 0.069, *P* = 0.628; belief relational: *r*(15) = 0.092, *P* = 0.617; belief representational: *r*(32) = 0.158, *P* = 0.373].

We additionally carried out an FSN analysis (Acar *et al.*, 2018) to further ensure the robustness of our findings against unpublished studies with null results in the ‘file drawer’ (e.g. driven by a bias towards publishing positive results). This approach entails investigating the effect of adding null-result experiments (i.e. null studies) to the original database of studies included in the meta-analysis (Acar *et al.*, 2018). Null-result experiments were created in R 3.6.1 (https://www.r-project.org), using Acar *et al.*’s (2018) R code and guidelines to match the real experiments in terms of sample size and number of foci reported, but with foci being distributed randomly across the brain. The resulting null experiments were then used to perform new meta-analyses addressing the FSN. The latter represents the number of noise studies (i.e. fMRI studies with non-significant results) that can be added to an ALE meta-analysis before a cluster is no longer significant. In practical terms, this approach entails assessing whether the FSN is below the lower bound (indicating non-robustness against publication bias) or above the upper bound (indicating that results are driven by a small number of hyper-influential studies). When the FSN lies between these boundaries, results can be considered sufficiently robust against the publication bias and driven by at least the desired minimum number of contributing studies. Following Acar *et al.* (2018), we pre-specified lower and upper boundaries for the FSN of each cluster based on the following considerations. A recent modelling approach to data included in the BrainMap database (http://brainmap.org/) suggests a rate of publication bias of up to 30% (i.e. up to 30 unpublished null studies per 100 published neuroimaging studies; Samartsidis *et al.*, 2019). We thus pre-specified that the FSN for each cluster should exceed a lower boundary of 30% of the real data (e.g. with 52 experiments, the minimum FSN is defined as 16). As to the upper boundary, each cluster is expected to be driven by at least 10% of the included studies. Accordingly, the upper boundary of the FSN was calculated per cluster as follows: ((number of studies contributing to a cluster)/0.1)) − (total number of studies included in the ALE meta-analysis). Only if the actual FSN obtained is between these two boundaries, the cluster can be assumed to be robust against both a potential file drawer effect and the effect of few hyper-influential experiments.

## Results

### Action representational processing

**Fig. 3.**
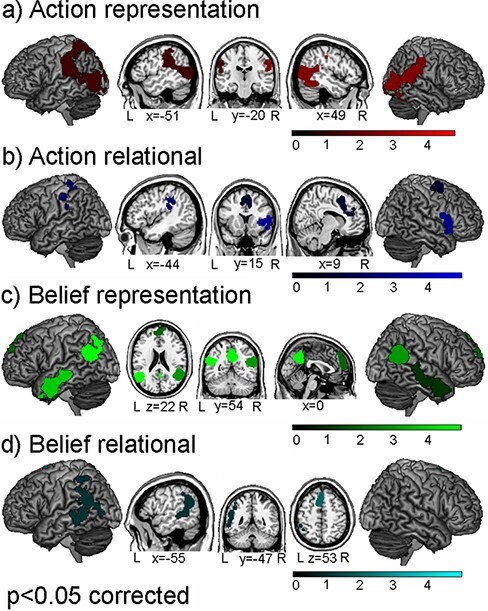
Brain activations associated with (A) action representation (red), (B) action relational (blue), (C) belief representation (green), and (D) belief relational (light blue) processes. L: left; R: right.

Representing another’s actions was associated with consistent activations in some of the key nodes of the mirror network, i.e. bilateral occipito-temporal (fusiform and inferior occipital gyri) and posterior lateral temporal (inferior, middle and superior temporal gyri) cortex , extending into the IPL, as well as in the left superior parietal lobule (Table 1; Figure 3A).

**Table 1.** The neural bases of action representational processing. From the left to right, the table reports the size (in mm^3^), stereotaxic coordinates of local maxima and anatomical labelling of the clusters which were consistently associated with action representational processing. The number of contributing experiments and the FSN for each cluster are also reported. For all clusters, the observed FSN lies between the two boundaries, meaning that results are sufficiently robust and supported by at least the desired minimum number of contributing studies.

Cluster number	Cluster size (mm^3^)	Brain region	*x*	*y*	*z*	Contributing experiments	FSN
1	62 008	Left supramarginal gyrus	−50	−34	28	51	16 < *x* < 458
		Left postcentral gyrus	−56	−40	46		
		Left superior parietal lobule	−14	−74	54		
		Left middle temporal gyrus	−60	−50	6		
		Left superior occipital gyrus	−22	−84	30		
		Left middle occipital gyrus	−24	−94	14		
		Left inferior occipital gyrus	−26	−94	−10		
2	40 056	Right postcentral gyrus	58	−18	40	46	16 < *x* < 408
		Right posterior superior temporal gyrus	62	−36	18		
		Right middle temporal gyrus	48	−68	2		
		Right fusiform gyrus	44	−48	−18		
		Right inferior occipital gyrus	40	−84	−2		

### Action relational processing

Action conflict monitoring was found to recruit the prefrontal cortex, including the right superior and middle frontal gyri alongside the anterior cingulate and supplementary motor cortex, and the left inferior parietal cortex, extending from the postcentral gyrus to the supramarginal gyrus and TPJ (Table 2; Figure 3B). Further activations involved the right insular/opercular cortex, extending rostrally into the inferior frontal gyrus and caudally into the superior temporal cortex.

**Table 2.** The neural bases of action relational processing. From the left to right, the table reports the size (in mm^3^), stereotaxic coordinates of local maxima and anatomical labelling of the clusters which were consistently associated with action relational processing. The number of contributing experiments and the FSN for each cluster are also reported. For all clusters, the observed FSN lies between the two boundaries, meaning that results are sufficiently robust and supported by at least the desired minimum number of contributing studies.

Cluster number	Cluster size (mm^3^)	Brain region	*x*	*y*	*z*	Contributing experiments	FSN
1	18 304	Medial SMA	4	16	52	7	4 < *x* < 55
		Left medial frontal gyrus	−8	16	42		
		Right superior frontal gyrus	24	−2	66		
		Right middle frontal gyrus	42	2	58		
		Right precentral gyrus	38	−10	62		
		Right middle cingulate gyrus	10	18	38		
		Right anterior cingulate	10	42	20		
2	11 264	Left precentral gyrus	−30	−26	56	5	4 <* x* <35
		Left postcentral gyrus	−38	−38	58		
		Left supramarginal gyrus	−50	−28	26		
		Left IPL	−56	−26	46		
		Left temporo-parietal junction	−42	−40	32		
		Left insula	−44	−32	22		
3	9624	Right insula	34	22	6	6	4 < *x* < 45
		Right inferior frontal gyrus	54	14	10		
		Right Rolandic operculum	62	6	16		
		Right superior temporal gyrus	52	14	−10		

### Belief representational processing

Representing others’ beliefs was associated with consistent midline activity in the dorsomedial prefrontal cortex (dmPFC) and precuneus, alongside bilateral clusters encompassing the TPJ and the anterolateral temporal cortex up to the temporal poles (Table 3; Figure 3C).

**Table 3.** The neural bases of belief representational processing. From the left to right, the table reports the size (in mm^3^), stereotaxic coordinates of local maxima and anatomical labelling of the clusters which were consistently associated with belief representational processing. The number of contributing experiments and the FSN for each cluster are also reported. For all clusters, the observed FSN lies between the two boundaries, meaning that results are sufficiently robust and supported by at least the desired minimum number of contributing studies.

Cluster number	Cluster size (mm^3^)	Brain region	*x*	*y*	*z*	Contributing experiments	FSN
2	15 952	Medial superior frontal gyrus	0	56	28	22	10 < *x* < 186
		Left superior frontal gyrus	−8	54	28		
4	13 568	Left middle temporal gyrus	−62	−22	−10	17	10 < *x* < 136
		Left temporal pole	−32	10	−36		
5	13 176	Medial precuneus	2	−54	34	26	10 < *x* < 226
		Left precuneus	−6	−50	56		
6	12 768	Left temporo-parietal junction	−50	−58	22	26	10 < *x* < 226
		Left middle temporal gyrus	−46	−76	30		
1	17 576	Right inferior temporal gyrus	52	6	−32	22	10 < *x* < 186
		Right middle temporal gyrus	54	0	−22		
		Right precentral gyrus	42	−2	−36		
3	13 720	Right middle temporal gyrus	58	−56	18	27	10 < *x* < 236
		Right temporo-parietal junction	52	−54	22		

### Belief relational processing

Mental conflict processing reflected in consistent dorsomedial prefrontal activity, alongside left hemispheric activations extending from the posterior lateral temporal cortex and TPJ to the inferior parietal cortex (Table 4; Figure 3D).

**Table 4.** The neural bases of belief relational processing. From the left to right, the table reports the size (in mm^3^), stereotaxic coordinates of local maxima and anatomical labelling of the clusters which were consistently associated with affective mentalizing. The number of contributing experiments and the FSN for each cluster are also reported. For all clusters, the observed FSN lies between the two boundaries, meaning that results are sufficiently robust and supported by at least the desired minimum number of contributing studies.

Cluster number	Cluster size (mm^3^)	Brain region	*x*	*y*	*z*	Contributing experiments	FSN
2	12 336	Left superior frontal gyrus	−6	18	48	12	5 < *x* < 103
		Medial superior frontal gyrus	4	36	42		
		Right middle cingulate gyrus	14	24	38		
		Left SMA	−6	8	58		
		Left middle cingulate gyrus	−4	24	34		
		Right SMA	8	8	66		
1	16 784	Left Temporo-parietal Junction	−56	−54	22	11	5 < *x* < 93
		Left IPL	−42	−46	44		
		Left middle temporal gyrus	−58	−46	8		
		Left middle occipital gyrus	−46	−74	4		

### Action relational and action representational processing

**Fig. 4.**
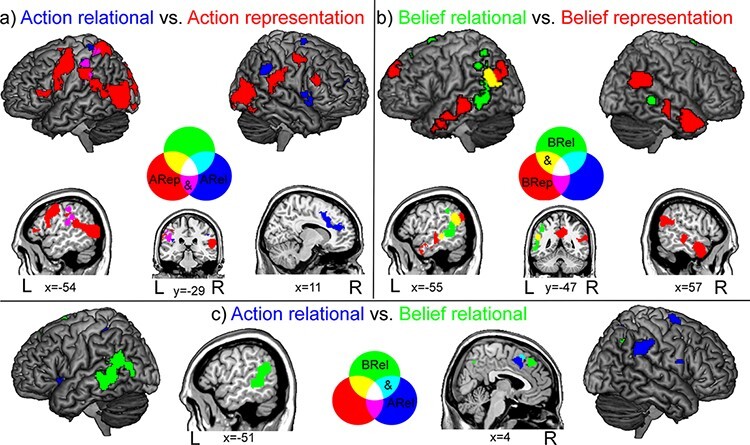
The figure depicts, with different colours, the common and specific brain structures across action representation and action relational processes (A), belief representation and belief relational processes (B) and action relational and belief relational processes (C). L: left; R: right.

A conjunction analysis unveiled common activity across action relational and action representational processing in the left IPL, supramarginal gyrus and TPJ (Table 5; Figure 4A). Direct comparisons highlighted specific activations for action representational processing in the precentral and inferior frontal gyri, alongside the superior and inferior parietal lobuli. Further activations for representing actions involved the occipito-temporal and posterior lateral temporal cortex bilaterally, extending into the superior temporal gyrus (Table 5; Figure 4A). Conversely, action relational processing was specifically associated with stronger frontal activity in the right anterior cingulate cortex and insula, and in the right superior and medial frontal gyri, alongside the IPL and the TPJ in the right hemisphere (Table 5; Figure 4A).

**Table 5.** Common and specific regions across action relational and action representational processing. From the left to right, the table reports the size (in mm^3^), stereotaxic coordinates of local maxima and anatomical labelling of the clusters which were commonly (top) and specifically (bottom) associated with action relational and action representational processing.

Cluster number	Cluster size (mm^3^)	Brain region	*x*	*y*	*z*
Action relational processing and representational processing					
1	7320	Left IPL	−38	−40	44
		Left supramarginal gyrus	−44	−38	32
		Left temporo-parietal junction	−54	−32	30
Action representational processing > relational processing					
3	11 880	Left precentral gyrus	−50	1	45
		Left inferior frontal gyrus	−56	14	14
		Left SMA	−18	2	64
5	2168	Right precentral gyrus	42	−6	46
6	1288	Right inferior frontal gyrus	60	32	18
7	608	Left inferior frontal gyrus	−50	34	10
12	304	Left postcentral gyrus	−42	−32	48
15	120	Left postcentral gyrus	−28	−36	50
4	3832	Left superior parietal lobule	−28	−52	68
8	512	Left superior parietal lobule	−34	−54	57
9	472	Left superior parietal lobule	−36	−56	58
10	456	Left superior parietal lobule	−38	−54	62
11	344	Left superior parietal lobule	−36	−60	66
13	256	Left superior parietal lobule	−34	−58	68
		Left IPL	−34	−58	50
14	152	Left IPL	−40	−48	54
1	28 240	Left middle temporal gyrus	−60	−50	2
		Left superior temporal gyrus	−55	−43	15
		Left middle occipital gyrus	−28	−88	2
		Left precentral gyrus	−58	−16	36
		Left precuneus	−24	−82	29
		Left inferior occipital gyrus	−26	−92	−6
		Left fusiform gyrus	−24	−94	−10
		Left IPL /left supramarginal gyrus	−56	−30	38
2	24 592	Right middle temporal gyrus	48	−68	4
		Right superior temporal gyrus	54	−26	16
		Right inferior occipital gyrus	34	−92	−2
		Right fusiform gyrus	36	−50	−10
		Right postcentral gyrus	60	−12	32
		Right inferior occipital gyrus	26	−90	−4
Action relational processing > representational processing					
1	7736	Right middle cingulate gyrus	6	20	34
		Right anterior cingulate	10	34	20
		Right medial frontal gyrus	14	54	18
		Right SMA	14	2	50
4	352	Right superior frontal gyrus	22	0	72
3	1440	Right angular gyrus/temporo-parietal junction	63	−40	36
5	312	Left postcentral gyrus/left IPL	−36	−32	64
6	120	Left IPL	−58	−26	50
2	3440	Right insula	44	16	−6
		Right putamen	28	14	2
		Right superior temporal gyrus	54	12	−2

### Belief relational and belief representational processing

Common activations to belief relational and representational processing were found in the medial superior frontal gyrus and in the left posterior sector of the middle temporal cortex, extending into the TPJ (Table 6; Figure 4B). Direct comparisons revealed bilateral activity specific to belief representational processing in the anterior cingulate and dmPFC, alongside the posterior lateral temporal cortex (extending into the right TPJ) and the anterolateral temporal cortex (extending into the temporal poles) (Table 6; Figure 4B). Instead, belief relational processing was specifically associated with activations in the middle cingulate/supplementary motor cortex and in the posterior middle temporal cortex, alongside the left IPL (including angular gyrus and supramarginal gyri) and the left TPJ (Table 6; Figure 4B).

**Table 6.** Common and specific regions across belief relational and representational processing. From the left to right, the table reports the size (in mm^3^), stereotaxic coordinates of local maxima and anatomical labelling of the clusters which were commonly (top) and specifically (bottom) associated with belief relational and representational processing.

Cluster number	Cluster size (mm^3^)	Brain region	*x*	*y*	*z*
Belief relational processing and representational processing					
3	32	Medial superior frontal gyrus	2	44	44
5	8	Medial superior frontal gyrus	0	42	46
1	4288	Left middle temporal gyrus	−50	−66	14
		Left temporo-parietal junction	−54	−52	28
2	600	Left middle temporal gyrus	−54	−30	−6
4	8	Left middle temporal gyrus	−62	−28	0
Belief representational processing > relational processing					
1	9736	Left superior frontal gyrus	−8	56	36
		Right anterior cingulate gyrus	12	52	11
		Medial frontal gyrus	0	54	22
		Right superior frontal gyrus	10	52	6
6	2912	Left middle temporal gyrus	−65	−20	−12
8	1080	Left middle temporal pole	−34	8	−36
		Left middle temporal gyrus	−56	8	−28
		Left inferior temporal gyrus	−55	0	−37
9	600	Left middle temporal gyrus	−62	−4	−24
		Left inferior temporal gyrus	−62	−8	−24
2	8448	Right middle temporal gyrus	54	4	−27
		Right inferior temporal gyrus	45	4	−35
3	7528	Right middle temporal gyrus/right temporo-parietal junction	53	−63	25
7	2584	Right superior temporal gyrus	56	−22	−2
		Right middle temporal gyrus	58	−24	−10
		Right inferior temporal gyrus	66	−20	−24
4	7000	Medial precuneus	3	−49	33
5	5816	Left angular gyrus	−44	−70	32
		Left middle occipital gyrus	−42	−78	40
Belief relational processing >representational processing					
1	10 168	Medial SMA	1	11	62
		Left SMA	−7	11	55
		Left superior frontal gyrus	−14	12	50
		Middle cingulate gyrus	−4	13	43
2	4216	Left middle temporal gyrus	−56	−42	−5
		Left temporo-parietal Junction	−58	−46	18
3	2000	Left IPL	−40	−50	50
4	608	Left supramarginal gyrus	−54	−36	36
5	544	Right middle temporal gyrus	62	−46	−3
6	376	Right precuneus	12	−68	54
7	328	Right angular gyrus	36	−56	42
		Right IPL	44	−62	44

### Belief relational and action relational processing

A conjunction analysis unveiled common activity across belief relational and action relational processing in the left posterior fronto-medial cortex, extending to the pre-supplementary motor area (SMA) and the right middle cingulate cortex, plus the left IPL (Table 7; Figure 4C). Direct comparisons highlighted specific activity for action relational processing in the middle cingulate cortex and the right precentral gyrus, alongside the fronto-insular and inferior parietal cortex bilaterally, with the additional right-hemispheric involvement of the TPJ (Table 7; Figure 4C). Instead, activations specific to belief relational processing were found in the dmPFC and precuneus, alongside the posterior middle temporal cortex in the left hemisphere and the TPJ bilaterally (Table 7; Figure 4C).

**Table 7.** Common and specific regions across belief relational and action relational processing. From the left to right, the table reports the size (in mm^3^), stereotaxic coordinates of local maxima and anatomical labelling of the clusters which were commonly (top) and specifically (bottom) associated with belief relational and action relational processing.

Cluster number	Cluster size (mm^3^)	Brain region	*x*	*y*	*z*
Belief relational processing and action relational processing					
1	2152	Pre-SMA	0	18	54
		Left posterior fronto-medial cortex	−8	16	44
2	256	Left IPL	−40	−42	44
3	232	Right middle cingulate gyrus	14	22	38
Action relational processing > belief relational Processing					
1	3104	Right medial frontal gyrus	12	15	50
		Right superior frontal gyrus	4	12	50
		Right middle cingulate gyrus	12	14	35
7	192	Right inferior frontal gyrus	60	8	20
		Right precentral gyrus	62	5	19
3	1152	Right superior frontal gyrus	22	6	64
8	168	Right superior parietal lobule	23	−63	60
9	160	Right anterior cingulate	14	41	15
10	120	Left postcentral gyrus	−38	−26	44
2	3008	Right supramarginal gyrus	60	−34	36
		Right IPL	60	−36	32
		Right temporo-parietal Junction	59	−34	28
4	1120	Left postcentral gyrus	−36	−36	52
		Left IPL	−36	−32	54
5	704	Left insula	−36	13	−11
6	392	Right anterior cingulate	10	32	22
Belief relational processing > action relational processing					
2	4264	Left superior frontal gyrus	−6	32	50
		Right superior frontal gyrus	8	34	47
		Medial superior frontal gyrus	−4	26	58
1	10 008	Left middle temporal gyrus	−55	−50	7
		Left middle temporal gyrus	−60	−47	−6
		Left middle occipital gyrus	−48	−71	9
		Left temporo-parietal junction	−51	−58	30
3	192	Right angular gyrus/right temporo-parietal junction	43	−58	43
4	120	Right precuneus	4	−62	44

### Publication bias

The FSN was always included between the two boundaries for all clusters (Tables 1**–**4), indicating that results are sufficiently robust against the publication bias, and supported by at least the desired minimum number of contributing studies.

## Discussion

The neurocognitive bases of understanding others’ behaviour have been typically framed in terms of representing their actions through the sensorimotor and premotor nodes of the mirror system (Rizzolatti and Craighero, 2004; Bonini, 2017), or their mental states via the medial prefrontal and TPJ sectors of the mentalizing network (Schurz *et al.*, 2014; Molenberghs *et al.*, 2016). Along with representing others’ motor and mental states, however, assessing their correspondence with our own ones might provide even more critical information for social understanding (Deschrijver and Palmer, 2020). This relational facet of social cognition entails monitoring social conflicts conveyed by action perception (Marsh *et al.*, 2016) and belief understanding (Bardi *et al.*, 2017). While converging evidence suggests the role of TPJ in social conflict monitoring (Deschrijver and Palmer, 2020), however, this proposal requires further supporting data (Schneider *et al.*, 2014; Campbell *et al.*, 2018). We addressed this issue by investigating the neural bases of representational and relational processing via coordinate-based meta-analyses of previous neuroimaging studies on false beliefs and action observation.

To ground our investigation in a detailed characterization of the neural bases of social understanding, we first assessed simple conditions and direct comparisons to confirm previous evidence about the neural bases of processing actions and beliefs, respectively. The former process engaged fundamental components of the action observation network (Cracco *et al.*, 2019), including temporo-parietal regions involved in processing multisensory information (Block *et al.*, 2013) and in sensorimotor transformations underpinning tool use (Orban, 2016) (Figure 3A). In contrast, representing others’ beliefs, regardless of one’s own ones, recruited the precuneus, TPJ, anterolateral temporal cortex and the rostral sector of dmPFC (Figure 3C). These regions have been associated with the multiple subprocesses of mentalizing, and particularly constructing and taking distinct perspectives, mediated by the precuneus (Hebscher *et al.*, 2018; Schurz *et al.*, 2020) and dmPFC (Ferrari *et al.*, 2016, 2017; Gamond and Cattaneo, 2016; Gamond *et al.*, 2017; Geiger *et al.*, 2019), respectively, via the retrieval of episodic and autobiographical memories. While the TPJ has been typically associated with transient mental inferences about people (Saxe and Kanwisher, 2003; Lamm *et al.*, 2007), the recruitment of temporo-parietal regions by tasks involving the reorientation of attention and a sense of agency (Sperduti *et al.*, 2011) led to suggest that they might support mentalizing via attentional reorienting (Dugué *et al.*, 2018). The latter hypothesis might help explaining the neural bases of relational processing, mostly involving regions adjacent to those associated with representation processing in the same domain, including distinct sectors of the TPJ.

In the action domain, relational processing indeed involved bilateral parietal/temporo-parietal areas and right-hemispheric premotor regions adjacent to, and partially overlapping with, the main nodes of the action observation network, alongside a posterior fronto-medial cluster encompassing the pre-SMA and dorsal anterior cingulate cortex (Figure 3B). The role of these regions in relational action processing might relate to previous reports of their engagement in imitation inhibition (Spengler *et al.*, 2009; Wang *et al.*, 2011). A functional distinction has been proposed for the TPJ and mPFC during imitation control, i.e. distinguishing between self- and other-generated actions and inhibiting the other-generated action to enforce the self-generated one (Brass *et al.*, 2009), respectively. The former function might support the TPJ role in processing social visuo-spatial conflict while concurrently generating a sense of agency, i.e. the feeling of being an entity performing an action localized in a specific space and perceiving the social world from this position and perspective (Ionta *et al.*, 2011).

In the belief domain, relational processing involved the left middle temporal cortex, extending into the TPJ and inferior parietal cortex, alongside the dmPFC (Figure 3D). While complementing previous fMRI (Cheung *et al.*, 2012) and electroencephalography (Chen *et al.*, 2012) evidence of TPJ involvement in false-belief understanding, these findings suggest that, also in the belief domain, this region might underpin relational processing by mediating self-other control processes. Distinguishing between self- and other-generated states indeed represents a crucial prerequisite for the subsequent mentalizing stage, i.e. inhibiting one’s own mental state to enhance others’ ones (Sowden and Shah, 2014; de Guzman *et al.*, 2016), likely involving the mPFC (Aron, 2007). In the false-belief task, this inhibitory process may support the inhibition of the true state of reality, also referred to as the ‘default’ state of belief representation (Leslie *et al.*, 2005). In this framework, false-belief performance is spontaneously driven by the true state of reality until participants detect the social conflict between their own (true) and the other/character’s (false) beliefs. Solving this social conflict, once this is made explicit by TPJ-mediated self-other control processes (Sowden and Shah, 2014; de Guzman *et al.*, 2016), might additionally require the dorsal mPFC for inhibiting the spontaneous tendency to respond according to one’s own beliefs (Rothmayr *et al.*, 2011).

Importantly, an interpretation of TPJ activity in terms of arbitration of social conflict between self- and other-related states fits with several relevant results regarding its role in other social–cognitive domains. For instance, the putative TPJ involvement in coding the difference between expected and observed outcomes (i.e. a ‘social prediction error’; Koster-Hale *et al.*, 2013; for a review, see Koster-Hale and Saxe, 2013) might also be interpreted as a social conflict processing between character’s belief on, and participant’s perception of, the subsequent real outcome (Deschrijver and Palmer, 2020). Moreover, in moral decision processing, TPJ activity is specifically associated with tasks generating a ‘social conflict’, such as attempted harm and accident, which in turn fits with the possible role of this region in processing the mismatch between character’s belief and participant’s perception of the real outcome (Deschrijver and Palmer, 2020).

While confirming the pattern highlighted by single conditions and direct comparisons, conjunction analyses within each domain provided further insights into the functional characterization of shared processes across representational and relational processing. A common activity in the action domain was found in the left temporo-parietal cortex (Figure 4A), whose involvement in encoding observed actions and invariant recognition of others’ actions (Ogawa and Inui, 2011, 2012) was found to depend, respectively, on the effector and the type of observed motor act regardless of the effector (Jastorff *et al.*, 2010). Instead, the common engagement of the left middle temporal cortex and TPJ by belief-related representational and relational processing (Figure 4B) might reflect their role in a superordinate requirement for mentalizing such as the storage of, and access to, socio-semantic knowledge (Lin *et al.,* 2018; Arioli *et al.*, 2020).

A focus on relational processing confirmed the engagement of the right TPJ and premotor cortex in action processing, and of the left TPJ and postero-temporal cortex in belief processing, respectively (Figure 4C). Moreover, both processes recruited the posterior fronto-medial cortex and the pre-SMA, in which adjacent clusters were selectively associated with action- and belief-related relational processing, respectively.

The observed lateralization of TPJ provides novel insights into the debate about the—possibly different—functional roles of its left- and right-hemispheric sectors. On the one hand, transcranial direct current stimulation (tDCS) of the left or right TPJ modulates performance on both inhibition of imitation and visual perspective-taking (Santiesteban *et al.*, 2015). Other findings, however, support their putative functional distinction in social cognitive processing. The left TPJ has been associated with tasks requiring to take a de-centred perspective, such as visual perspective-taking (Schurz *et al.*, 2013), false-belief processing (Hartwright *et al.*, 2012; Arora *et al.*, 2017) and strategic decision-making with other humans (Ogawa and Kameda, 2019). Instead, inhibition of imitation might represent a superordinate function of the right TPJ (Brass *et al.*, 2005; Spengler *et al.*, 2009), with its response being modulated by the perception of human agency (i.e. action monitoring; Ogawa and Kameda, 2019; also in the condition of visual deprivation; Arioli *et al.*, 2021b) and by the presence of animacy cues leading to believe that an interaction partner is human (Klapper *et al.*, 2014). In line with this hypothesis, imitation performance is influenced by TMS-mediated modulation of the right TPJ (Hogeveen *et al.*, 2014; Sowden and Catmur, 2015).

This view of TPJ engagement in perspective-taking has been refined in distinct directions. On the one hand, this role seems limited to social inferences such as ‘considering the perspective of another agent’ rather than ‘the perspective of an arrow’ (Schurz *et al.*, 2015). Moreover, the observed involvement of the posterior part of TPJ in processing self-other distinctions at the mental-state level (Quesque and Brass, 2019) fits with previous evidence that this sector underpins mentalizing and internally directed attention by deactivating the neighbouring anterior portion (Kubit and Jack, 2013). By showing that only the posterior part of the right TPJ is associated with belief conflict processing, while the anterior part is involved in action conflict monitoring, our results suggest that previous conflicting results concerning lateralization effects might be biased by targeting its different functional subdivisions. Altogether, these findings suggest that the TPJ role in processing self-other distinctions supports the monitoring of social conflicts conveyed by neural representations of both actions (Marsh *et al.*, 2016) and mental states (e.g. beliefs; Bardi *et al.*, 2017) involving the premotor and postero-temporal cortex, respectively.

Adjacent, and partially overlapping, activations for action- and belief-related relational processing were found in the pre-SMA (Cona and Semenza, 2017) and in the posterior fronto-medial cortex (Gamond and Cattaneo, 2016* *) (cyan in Figure 4C). In addition to action monitoring (Bonini *et al.*, 2014), the activity of pre-SMA has been associated with social evaluation (e.g. Wen *et al.*, 2017), probably reflecting both sensorimotor and emotional aspects of social interactions, such as contagiousness of laughter (McGettigan *et al.*, 2015) or empathic response (Akitsuki and Decety, 2009).

Among the several functions that have been ascribed to the posterior fronto-medial cortex (e.g. Simmonds *et al.*, 2008; Dodell-Feder *et al.*, 2011; Ferrari *et al.*, 2017), executive functioning (Wager *et al.*, 2004; Alvarez and Emory, 2006; Houdé *et al.*, 2010) supports inhibition control (Wade *et al.*, 2018) and cognitive control (Ridderinkhof *et al.*, 2004), in turn representing a crucial prerequisite for monitoring social conflicts (Wake *et al.*, 2019) both in the action (Heyes, 2011) and belief (Aichhorn *et al.*, 2009; Döhnel *et al.*, 2012; Özdem *et al.*, 2019) domains. In particular, the medial frontal cortex is considered to manage the social conflict between self- and other-generated actions, once these are distinguished by the TPJ, enforcing the former and the associated first-person intention (Brass *et al.*, 2009; Ninomiya *et al.*, 2018). In the action domain, an inherent aspect of this process is represented by the top-down modulation of imitation (Wang *et al.*, 2011), possibly explaining the disrupted inhibition of imitation displayed by patients with frontal lesions (Brass *et al.*, 2003; Spengler *et al.*, 2010), and their tendency to automatically imitate even when they are clearly instructed to not do so (Lhermitte *et al.*, 1986). The present evidence that the posterior fronto-medial cortex is also involved in belief conflict monitoring might help explain its role in mentalizing by subserving the representation of intentional states for both self and other (Amodio and Frith, 2006). Its activation during the outcome phase of false-belief tasks, when expectations about the object location can be violated (Bardi *et al.*, 2017), might indeed reflect its role in managing ‘relational’ social conflicts. Hartwright *et al.* (2012) reported the activation of frontal executive-control regions during false- *vs* true-belief processing, reflecting the need to inhibit self-perspective. Overall, mentalizing judgements would be thus supported by multiple processes such as distinguishing and switching between one’s own and another’s beliefs through the TPJ and posterior fronto-medial cortex, respectively (Gunia *et al.*, 2021). Interestingly, a similar pattern of brain activity has been also reported during implicit false-belief processing (Kovács *et al.*, 2014; Naughtin *et al.*, 2017). Causal supporting evidence for this proposal comes from neuromodulation studies showing either up- and down-regulation of egocentric biases, associated with a consistent decrease and increase of the influence of another’s mental or visuo-spatial perspective, after low-frequency repetitive TMS (Schuwerk *et al.*, 2014) or anodal tDCS (Martin *et al.*, 2017), respectively. According to the authors, stimulating the posterior fronto-medial cortex might enhance the integration of information about another’s mental (Schuwerk *et al.*, 2014) or visuo-spatial (Martin *et al.*, 2017) perspective into one’s own. While the latter hypothesis requires further supporting evidence, the data collected so far suggest that the posterior fronto-medial cortex participates in social conflict monitoring both by enhancing the required belief or action responses, and by inhibiting the automatic tendency to report the true belief or to imitate (Bardi *et al.*, 2009, 2017; Wake *et al.*, 2019; Gunia *et al.*, 2021).

Methodological guidelines for coordinate neuroimaging meta-analyses led to select only studies performing the whole-brain analysis (Müller *et al.*, 2018). It is important to stress, however, that similar results on the role of the TPJ and posterior fronto-medial cortex in processing social conflict have been also reported by studies in which strong a priori hypotheses justified ROI analyses or SVC procedures [e.g. Boccadoro *et al.*, 2019; Klapper *et al.*, 2014; Nijhof *et al.*, 2018; see Schurz *et al.* (2014) for a meta-analysis based on ROIs].

Considering Deschrijver and Palmer’s (2020) hypothesis, our rigorous approach provided results supporting the role of the left TPJ in belief conflict processing, through the distinction between self- and other-related states, rather than to belief representation *per se*. However, unlike Deschrijver and Palmer’s (2020) predictions and Hartwright *et al.*’s (2012) findings, we did not provide conclusive evidence for the involvement of the right TPJ in false-belief processing. Moreover, we also reported unexpected evidence , such as the right TPJ activity in association with action (*vs* belief) conflict processing, and the role of the posterior fronto-medial cortex in both action and belief conflict processing, through the inhibition of conflictual representations.

Importantly, unveiling the properties and neural bases of relational processing might help extend current perspectives of impaired social cognition and communication (e.g. Santiesteban *et al.*, 2012). Based on the considerable individual variability in perceiving or interpreting even a same event (Wilhelm *et al.*, 2010; Miller and Saygin, 2013), social communication and interactions may depend on tracking how well present information about others aligns with one’s own perspective even more than inferring their states. Impairments in detecting, processing or solving experiential differences are thus expected to reflect in altered social communication and/or interactions. In line with this hypothesis, several populations—including young children (Priewasser *et al.*, 2018) and non-human primates (Martin and Santos, 2016)—display altered performance in false-belief tasks despite the normal understanding of true-belief situations. This evidence confirms that generating another’s specific representation of the world in false-belief paradigms is qualitatively different from, and more difficult than, attributing one’s own one like in true-belief situations (Martin and Santos, 2014; Martin and Santos, 2016). Indeed, growing evidence suggests that the multifaceted pattern of social deficits observed in autism may reflect subtle issues with monitoring social conflict, rather than altered representational abilities or ‘mindblindness’ altogether (Burnside *et al.*, 2017; Nijhof *et al.*, 2018). This hypothesis has been recently assessed with an implicit false-belief task (Deschrijver *et al.*, 2016) merely requiring to respond when a visual target is detected while holding constant belief manipulations as in the Sally–Anne task. It might be speculated that RTs reflect participants’ belief about the stimulus location (Martin and Santos, 2014; Bardi *et al.*, 2017). Instead, another person’s belief that the target would be present can speed up target detection, even though participants themselves had been convinced that the target would not be present (Kovacs *et al.*, 2010; Deschrijver *et al.*, 2016). In this context, an account of autism centred on impaired mentalizing, and thus on the lack of belief representations (Frith, 2001), would not be expected to predict this contribution to detection performance. Instead, strong autistic traits were associated with slowed responses when participants—after being convinced of the stimulus absence—were informed about another’s, opposite, belief. This finding suggests a preserved representation of the other person’s belief, associated with altered monitoring/resolution of social conflicts between that representation and one’s own one, particularly when the latter must be expressed (Deschrijver *et al.*, 2016). This conclusion is strengthened by an explicit experimental paradigm showing that the impact of others’ false beliefs is enhanced, in autistic individuals more than controls, when the task requires to verbalize their own mental state (Sommer *et al.*, 2018). While further evidence is required to evaluate the contribution of altered relational processing, these data at least suggest that autistic individuals’ social impairment is not fully explained by a ‘representational’ framework, i.e. by difficulties in representing others’ intentions—be it in terms of mental states or actions—*per se*. These data therefore suggest that autistic individuals’ social impairment might be better explained by altered relational, than representational, processing and that the autistic brain might fail to monitor the social conflict between one’s own and others’—properly represented—actions or mental states. This hypothesis fits with the evidence that, at the neural level, the difficulties of autistic patients with social conflict processing are associated with decreased TPJ activity (Nijhof *et al.*, 2018).

This hypothesis has already been translated into potential treatment strategies, with participants being trained to either imitate or inhibit imitation, and thus to either represent another’s actions or monitor action conflict (Santiesteban *et al.*, 2012). Within a ‘relational’ framework, training action conflict monitoring (rather than action representation) was expected to improve mentalizing task comparing incongruent with congruent social conditions. Training to monitor action conflict, compared with training to represent others’ actions, was indeed associated with improved performance on this mentalizing task. By showing a transfer from the trained monitoring of action conflict to the monitoring of conflictual mental (rather than action) representations, these results suggest the existence of (at least partially) common underlying processes of social conflict monitoring transcending single domains. These data support the notion that imitation–inhibition training enhances self-other distinction processing, which in turn facilitates improved performance on both the imitation–inhibition and mentalizing tasks. Our results support this view by showing a common engagement of the posterior fronto-medial cortex for both action- and belief-related relational processing, alongside domain-specific sectors in adjacent posterior fronto-medial regions and in the left- and right-hemispheric TPJ sectors. Overall, this pattern of findings highlights the potential effect of training social conflict monitoring in rehabilitation protocols for autistic patients, possibly in conjunction with neuromodulation techniques targeting the TPJ and/or posterior fronto-medial regions shown by the present meta-analytic results.

### Limitations

A limitation of our work is the selection of “suboptimal” contrasts to study belief representational processing, i.e. (i) true belief > control non-mental condition and (ii) false belief > false photograph. Neither contrasts, indeed, are free from limitations. The former contrast allows to control for the structure of the stimuli, but in the target “true-belief” condition participants might rely only on their own mental states, possibly attributed to the story character, without necessarily generating a representation of the character’s mental states (Martin and Santos, 2016). The second contrast, using the “false-belief” condition as a target condition, addresses not only mental-state representation but also social conflict. There is no evidence that the “false-photograph” condition (with perceptual conflict) can control for social conflict. At this stage, how to address the representation of mental states, while isolating this process from the representation of one’s own mental states and from social conflict between one’s own and others’ mental states, remains controversial.

A partial solution is provided by Hartwright *et al.*’s (2012) study. In this experiment, as in a classical Sally–Anne false-belief task, participants are asked to predict which boxes a character would open based on the scenario presented. Each scenario entails three randomly ordered statements, concerning character’s belief, character’s desire and reality (about the location of the desired object). Such randomized order ensured participants’ encoding of the character’s true belief on at least 50% of trials in which they did not already know the object’s true location. This design therefore allowed to address the weakness of several studies in which participants could provide the correct answer, even when ignoring a character’s beliefs on true-belief trials, by merely relying on their own knowledge of reality. Isolating ‘mental-state representation’ would require comparing this type of the ‘true-belief’ condition with a non-mental control condition.

## Conclusion

Our work provides novel neural evidence showing specific brain activity for belief and action conflict processing. This evidence supports the unique status of relational processing, i.e. the ability to evaluate whether, how and how much others’ states (mis)match with our own ones, which might represent an even more important prerequisite for effective social communication and interactions than representing those states via the action observation or mentalizing network. The present findings suggest that this process involves adjacent sectors of the posterior fronto-medial cortex, differing in their selectivity to the action or belief domains, whereby social conflict processing is supported by the inhibition of conflictual representations (Santiesteban *et al.*, 2012), as well as the left and right TPJ, likely contributing to self-other differentiation for mental and action states, respectively. These findings pave the way for further studies on the main building blocks of normal and pathological social cognition, and for the design of rehabilitative treatment protocols based on their neurocognitive characterization.

## Supplementary Material

nsad003_Supp

## Data Availability

Data from this study are available from the corresponding author upon request.
