# Supplementary material for: Relational vs representational social cognitive processing: a coordinate-based meta-analysis of neuroimaging data
Source: Soc Cogn Affect Neurosci. 2023 Jan 25;18(1):nsad003. doi: 10.1093/scan/nsad003 (PMC9976764; doi:10.1093/scan/nsad003)
Supplement: nsad003_Supp [file nsad003_supp.zip › scan-21-216-File002.docx]

**Supplementary File:**

**Supplementary Tables:**

| **N** | **Author** | **Year** | **Title** | **Sub (mean age)** | **Stimuli** | **Task** | **Contrast** | **Foci** |
| --- | --- | --- | --- | --- | --- | --- | --- | --- |
| 1 | Abraham et al | 2008 | Minds, persons, and space: An fMRI investigation into the relational complexity of higher-order intentionality | 17 sub (mean age: 25.65) | written sentence | comprehension task (yes-no questions) | true belief > control (non-mental) | 38 |
| 2 | Aichhorn et al | 2009 | Temporo-parietal junction activity in theory-of-mind tasks: falseness, beliefs, or attention | 15 sub (mean age: 24) | verbal vignettes | comprehension task (answer questions) | false belief > perspective difference | 9 |
| 3 | Alderson-Day et al | 2016 | The brain’s conversation with itself: neural substrates of dialogic inner speech | 21 sub (mean age: 24.38) | cartoon stories | choose the end of the story | true belief > physical | 10 |
| 4 | Andrews-Hanna et al | 2014 | Contributions of episodic retrieval and mentalizing to autobiographical thought: evidence from functional neuroimaging, resting-state connectivity, and fMRI meta-analyses | 35 sub (mean age:21.8) | short stories | comprehension task (answer questions) | false belief > false photograph | 9 |
| 5 | Cheung et al | 2012 | False belief and verb non-factivity: a common neural basis? | 20 sub (mean age: 23.5) | short sentences and visual images | comprehension task (answer questions) | true belief > control | 11 |
| 6 | Contreras et al | 2013 | Common Brain Regions with Distinct Patterns of Neural Responses during Mentalizing about Groups and Individuals | 20 sub (mean age: 22) | photographs depicting group of people | photograph judgment task (mental or physical) | true belief > physical | 14 |
| 7 | Corradi-Dell'Acqua et al | 2014 | Cognitive and affective theory of mind share the same local patterns of activity in posterior temporal but not medial prefrontal cortex | 46 sub (age range: 18-31) | short written stories | judgment task (mentalizing, emotions, physical) | false belief > photos | 9 |
| 8 | Cracco et al | 2020 | Early interpersonal trauma reduces temporoparietal junction activity during spontaneous mentalizing | 31 sub (mean age: 36.51) | visual movies | Implicit ToM task | false belief > false photograph | 18 |
| 9 | De Coster et al | 2019 | Neural and behavioral effects of oxytocin administration during theory of mind in schizophrenia and controls: a randomized control trial | 25 sub (mean age: 27.96) | written short stories | comprehension task (true/false questions) | false belief > false photographs | 5 |
| 10 | Dodell-Feder et al | 2014 | The neural basis of theory of mind and its relationship to social functioning and social anhedonia in individuals with schizophrenia | 18 sub (mean age: 32.4) | short written stories | answer true/false questions | false belief > false photograph | 18 |
| 11 | Dodell-Feder et al | 2011 | fMRI item analysis in a theory of mind task | 62 sub (mean age:22) | written stories | answer true/false questions | false belief > false photograph | 27 |
| 12 | Döhnel et al | 2012 | Functional activity of the right temporo-parietal junction and of the medial prefrontal cortex associated with true and false belief reasoning | 18 sub (mean age: 24.72) | non-verbal stories | comprehension task (answer questions) | false and true belief > control conditions | 8 |
| 13 | Gweon et al | 2012 | Theory of Mind Performance in Children Correlates With Functional Specialization of a Brain Region for Thinking About Thoughts | 8 sub (mean age: 21.5) | written stories | comprehension task (yes/no questions) | true mental > physical | 6 |
| 14 | Hartwright et al | 2015 | The special case of self-perspective inhibition in mental, but not non-mental, representation | 21 sub (mean age: 22) | verbal vignettes | comprehension task (true/false questions) | false belief > false physical | 13 |
| 15 | Hartwright et al | 2012 | Multiple roles for executive control in belief–desire reasoning: Distinct neural networks are recruited for self perspective inhibition and complexity of reasoning | 19 sub (mean age: 25) | statements + pictures | comprehension task (answer questions) | false belief > false photos | 7 |
| 16 | Jacoby et al | 2016 | Localizing Pain Matrix and Theory of Mind networks with both verbal and non-verbal stimuli | 20 sub (mean age: 25.3) | written stories | comprehension task (true/false questions) | false belief > false photographs | 11 |
| 17 | Jenkins et al | 2014 | The Neural Bases of Directed and Spontaneous Mental State Attributions to Group Agents | 19 sub (mean age:21) | verbal narratives | comprehension task (true/false questions) | false belief > photos | 9 |
| 18 | Jimura et al | 2010 | Temporal pole activity during understanding other persons' mental states correlates with neuroticism trait | 34 sub (mean age: 20-28) | written phrases | comprehension task (answer questions) | false belief > control for false belief | 4 |
| 19 | Kandylaki et al | 2015 | Processing of false belief passages during natural story comprehension: An fMRI study | 20 sub (mean age: 24.3) | auditory stories | comprehension task (answer questions) | false belief > control for false belief | 17 |
| 20 | Kobayashi et al | 2006 | Cultural and linguistic influence on neural bases of 'Theory of Mind': an fMRI study with Japanese bilinguals | exp1: 16 sub (age range 18-39) | written stories | comprehension task (answer questions) | false belief > control for false belief (monolingual) | 5 |
|  |  |  |  | exp2: 16 sub (age range 18-39) | written stories | comprehension task (answer questions) | false belief > control for false belief (bilingual) | 8 |
| 21 | Lee et al | 2016 | Neural Correlates of Belief and Emotion Attribution in Schizophrenia | 14 sub (mean age: 41.80) | visual vignettes | comprehension task (answer questions) | false belief > false photograph | 8 |
| 22 | Lee et al | 2011 | Theory of mind in schizophrenia: exploring neural mechanisms of belief attribution | 13 sub (mean age: 42.5) | vignettes | comprehension task (answer questions) | false belief > false photos | 16 |
| 23 | Mitchell | 2008 | Activity in right temporo-parietal junction is not selective for theory-of-mind | 20 sub (mean age: 23.0) | written vignettes | comprehension task (answer questions) | false belief > false photographs | 3 |
| 24 | Moran et al | 2012 | Social-cognitive deficits in normal aging | 28 sub (mean age: 23.0) | written stories | comprehension task (answer questions) | false belief > false photos | 34 |
| 25 | Oliver et al | 2018 | Greater involvement of action simulation mechanisms in emotional vs cognitive empathy | 36 sub (mean age: 21.5) | written stories | comprehension task (answer questions) | false belief > false photos | 9 |
| 26 | Özdem et al | 2017 | The overlap between false belief and spatial reorientation in the temporo-parietal junction: The role of input modality and task | 20 sub (mean age:21.60) | geometrical shapes | comprehension task (answer questions) | false belief > false photos | 5 |
| 27 | Saxe et al | 2006a | Reading minds versus following rules: dissociating theory of mind and executive control in the brain | 12 sub (age range: 18-26) | written stories | comprehension task (answer questions) | false belief > false photos | 8 |
| 28 | Saxe et al | 2006b | It’s the Thought That Counts Specific Brain Regions for One Component of Theory of Mind | 12 sub (age range:19-26) | written stories | press the button when she/he finished reading | false belief > false photos | 9 |
| 29 | Specht et al | 2018 | The functional and structural asymmetries of the superior temporal sulcus | 18 sub (mean age: 25.7) | cartoon stories | select the end of the story | true belief > control | 15 |
| 30 | Völlm et al | 2006 | Neuronal correlates of theory of mind and empathy: a functional magnetic resonance imaging study in a nonverbal task. | 13 sub (mean age: 24.9) | comic strips | select the end of the story | true belief > physical | 13 |
| 31 | Wang et al | 2015 | Dimensional schizotypy and social cognition: an fMRI imaging study | 52 sub (mean age: 19.3) | comic strips | select the end of the story | true belief > physical | 7 |
| 32 | Wysocka et al | 2020 | Processing false beliefs in preschool children and adults: Developing a set of custom tasks to test the theory of mind in neuroimaging and behavioral research | 13 sub (mean age: 29) | visual cartoons | comprehension task (answer questions) | true and false belief > control condition | 13 |
| 33 | Young et al | 2010 | What gets the attention of the temporo-parietal junction? An fMRI investigation of attention and theory of mind | 17 sub (age range: 18-31) | written stories | comprehension task (answer questions) | false belief > false photos | 9 |
|  |  |  |  | **TOT SUB: 769** |  |  |  | **TOT FOCI: 405** |

**Supplementary Table 1**: Overview of the 33 studies included in the meta-analysis on the neural bases of belief representational processing. N: progressive study number; Sub: subjects

| **N** | **Author** | **Year** | **Title** | **Sub (mean age)** | **Stimuli** | **Task** | **Contrast** | **Foci** |
| --- | --- | --- | --- | --- | --- | --- | --- | --- |
| 1 | Abraham et al | 2010 | Matching mind to world and vice versa: Functional dissociations between belief and desire mental state processing | 22 sub (mean age: 26.14) | written sentence | comprehension task (yes-no questions) | false belief > true belief | 17 |
| 2 | Aichhorn et al | 2009 | Temporo-parietal junction activity in theory-of-mind tasks: falseness, beliefs, or attention | 21 sub (mean age: 24) | verbal vignettes | comprehension task (answer questions) | false belief > true belief | 8 |
| 3 | Bardi et al | 2017 | Brain activation for spontaneous and explicit false belief tasks overlaps: new fMRI evidence on belief processing and violation of expectation | 22 sub (mean age: 22) | movies consisted of two phases | spontaneous and the explicit mentalizing task | false belief > true belief | 2 |
| 4 | Cracco et al | 2020 | Early interpersonal trauma reduces temporoparietal junction activity during spontaneous mentalizing | 31 sub (mean age: 36.51) | visual movies | Implicit ToM task | false belief > true belief | 17 |
| 5 | Döhnel et al | 2017 | An fMRI study on the comparison of different types of false belief reasoning: False belief-based emotion and behavior attribution | 22 sub (mean age: 25.27) | cartoon stories | comprehension task (answer questions) | false belief > true belief | 20 |
| 6 | Döhnel et al | 2012 | Functional activity of the right temporo-parietal junction and of the medial prefrontal cortex associated with true and false belief reasoning | 18 sub (mean age: 24.72) | non-verbal stories | comprehension task (answer questions) | false belief > true belief | 9 |
| 7 | Grèzes et al | 2004 | Inferring false beliefs from the actions of oneself and others: an fMRI study | 6 sub (mean age: 25-39) | videos of actors | comprehension task (answer questions) | false belief > true belief | 10 |
| 8 | Hartwright et al | 2014 | Representation, Control, or Reasoning? Distinct Functions for Theory of Mind within the Medial Prefrontal Cortex | 20 sub (mean age: 21) | computerized virtual game | prediction task | false belief > true belief | 24 |
| 9 | Hooker et al. | 2008 | Mentalizing about emotion and its relationship to empathy | 20 sub (mean age: 21) | videos of human avatars | emotion inference tasks | false belief > true belief | 17 |
| 10 | Özdem et al | 2019 | The neural representation of mental beliefs held by two agents | 25 sub (age range: 18-28) | animated video clips | comprehension task (answer questions) | false belief > true belief | 3 |
| 11 | Rothmayr et al | 2011 | Common and distinct neural networks for false-belief reasoning and inhibitory control | 12 sub (mean age: 23.7) | non-verbal cartoon stories | comprehension task (answer questions) | false belief > true belief | 13 |
| 12 | Schuwerk et al | 2014 | Functional Activity and Effective Connectivity of the Posterior Medial Prefrontal Cortex During Processing of Incongruent Mental States | 21 sub (mean age: 23.3) | cartoon videos | comprehension task (answer questions) | false belief > true belief | 18 |
| 13 | Sommer et al | 2010 | Modulation of the cortical false belief network during development | 20 sub (mean age: 32.7) | cartoon stories | comprehension task (answer questions) | false belief > true belief | 5 |
| 14 | Sommer et al | 2018 | False Belief Reasoning in Adults with and without Autistic Spectrum Disorder: Similarities and Differences | 15 sub (mean age: 29.9) | cartoon stories | comprehension task (answer questions) | false belief > true belief | 6 |
| 15 | Sommer et al | 2007 | Neural correlates of true and false belief reasoning | 16 sub (mean age: 26) | non verbal cartoons stories | comprehension task (answer questions) | false belief > true belief | 8 |
| 16 | van der Meer et al | 2011 | Inhibit yourself and understand the other: Neural basis of distinct processes underlying Theory of Mind | 19 sub (mean age: 21.6) | short movie clips | comprehension task (answer questions) | false belief > true belief | 23 |
| 17 | Wysocka et al | 2020 | Processing false beliefs in preschool children and adults: Developing a set of custom tasks to test the theory of mind in neuroimaging and behavioral research | 13 sub (mean age: 29) | visual cartoons | comprehension task (answer questions) | false belief > true belief | 2 |
|  |  |  |  | **TOT SUB: 323** |  |  |  | **TOT FOCI: 202** |

**Supplementary Table 2**: Overview of the 17 studies included in the meta-analysis on the neural bases of belief relational processing. N: progressive study number; Sub: subjects

| **N** | **Author** | **Year** | **Title** | **Sub (mean age)** | **Stimuli** | **Task** | **Contrast** | **Foci** |
| --- | --- | --- | --- | --- | --- | --- | --- | --- |
| 1 | Abdollahi et al | 2012 | Common and segregated processing of observed actions in human SPL | 15 sub (mean age: 24) | videos of various actions, scrambled controls | passive viewing | action observation > scrambled control | 66 |
| 2 | Adamovich et al | 2009 | A virtual reality-based system integrated with fmri to study neural mechanisms of action observation-execution: a proof of concept study | 13 sub (mean age: 27.7) | hand actions and moving objects in VR | passive viewing | action observation > object control | 26 |
| 3 | Arioli et al | 2018 | Affective and cooperative social interactions modulate effective connectivity within and between the mirror and mentalizing systems | 36 sub (mean age: 24.9) | videos of goal-directed interactions, landscape controls | respond to landscape images | action observation > landscape control | 14 |
| 4 | Bach et al | 2010 | On the role of object information in action observation: an fMRI study | 10 sub (age range: 18-34) | sequences of various actions, object controls | detect mismatches | action observation > object control | 5 |
| 5 | Baumgaertner et al | 2007 | Polymodal conceptual processing of human biological actions in the left inferior frontal lobe | 19 sub (mean age: 27.3) | videos of daily life actions, object controls | acceptability judgment | action observation > object control | 13 |
| 6 | Biagi et al | 2010 | Anterior intraparietal cortex codes complexity of observed hand movements | 20 sub (mean age: 31) | videos of various actions, static controls | passive viewing | action observation > static control | 22 |
| 7 | Buccino et al | 2001 | Action observation activates premotor and parietal areas in a somatotopic manner: an fMRI study | 12 sub (age range: 25-38) | videos of mouth/hand/foot actions, static controls | passive viewing | action observation > static control | 28 |
| 8 | Cheng et al | 2007 | Motivation modulates the activity of the human mirror-neuron system | 20 sub (age range: 19-28) | videos of grasping actions, object controls | passive viewing | action observation > object control | 37 |
| 9 | Corbo et al | 2017 | Observing Others Speak or Sing Activates Spt and Neighboring Parietal Cortex | 24 sub (mean age: 25) | videos of hand/mouth actions, scrambled controls | passive viewing | action observation > scrambled control | 19 |
| 10 | De Vega et al | 2014 | Action sentences activate sensory motor regions in the brain independently of their status of reality | 19 sub (mean age: 24) | videos of various actions, object controls | passive viewing | action observation > static control | 17 |
| 11 | Di Cesare et al | 2014 | The neural correlates of 'vitality form' recognition: an fMRI study | 19 sub (mean age: 24.25) | videos of dyadic interactions, static controls | discern action/vitality form | action observation > static control | 32 |
| 12 | Di Dio et al | 2013 | The neural correlates of velocity processing during the observation of a biological effector in the parietal and premotor cortex | exp1: 14 sub (mean age: 23.5) | videos of reaching actions, static controls | passive viewing | action observation > static control | 17 |
|  |  |  |  | exp2: 16 sub (mean age: 23.6) | videos of a moving arm, static controls | passive viewing | action observation > static control | 17 |
| 13 | Engel et al | 2008 | How moving objects become animated: the human mirror neuron system assimilates non-biological movement patterns | 18 sub (mean age: 23) | videos of hand actions, object and static controls | passive viewing | action observation > object/static control | 26 |
| 14 | Fadiga et al | 2006 | Language in shadow | 10 sub (mean age: 23) | videos of hand actions, static controls | passive viewing | action observation > static control | 30 |
| 15 | Ferri et al | 2015 | The organization of the posterior parietal cortex devoted to upper limb actions: An fMRI study | 28 sub (mean age: 23) | videos of various actions, scrambled controls | passive viewing | action observation > scrambled control | 43 |
| 16 | Filimon et al | 2007 | Human cortical representations for reaching: mirror neurons for execution, observation, and imagery | 16 sub (age range: 19-48) | videos of reaching actions, object controls | passive viewing | action observation > object control | 14 |
| 17 | Georgescu et al | 2014 | Perceiving nonverbal behavior: neural correlates of processing movement fluency and contingency in dyadic interactions | 21 sub (mean age: 26.86) | videos of animated dyadic actions, scrambled controls | judge movement fluency | action observation > scrambled control | 20 |
| 18 | Grèzes et al | 2006 | Perceiving fear in dynamic body expressions | 16 sub (mean age: 25) | videos and images of door opening, scrambled controls | detect odd trials | action observation > scrambled control | 17 |
| 19 | Grosbras et al | 2005 | Brain networks involved in viewing angry hands or faces | 20 sub (mean age: 28.6) | videos of hand actions, abstract controls | passive viewing | action observation > abstract control | 57 |
| 20 | Hamilton et al | 2006 | Where does your own action influence your perception of another person's action in the brain? | 19 sub (age range: 18-35) | videos of lifting actions, object controls | weight judgment/flicker counting | action observation > object control | 12 |
| 21 | Hamzei et al | 2003 | The human action recognition system and its relationship to Broca's area: an fMRI study | 12 sub (age range: 20-30) | videos of drinking actions, no action controls | passive viewing | action observation > no action control | 3 |
| 22 | Heitger et al | 2012 | Cortical regions involved in the observation of bimanual actions | 19 sub (mean age: 23.4) | videos of hand actions, moving object controls | passive viewing | action observation > object control | 50 |
| 23 | Iacoboni et al | 2005 | Grasping the intentions of others with one’s own mirror neuron system | 23 sub (mean age: 26.3) | videos of grasping actions, object controls | infer intentions | action observation > object control | 36 |
| 24 | Iseki et al | 2008 | Neural mechanisms involved in mental imagery and observation of gait | 16 sub (mean age: 34.3) | videos of gait movement, scrambled controls | passive viewing | action observation > scrambled control | 11 |
| 25 | Jastorff et al | 2016 | Seeing biological actions in 3D: An fMRI study | 21 sub (mean age: 23) | 3D point-light action videos, scrambled controls | discern 2D and 3D videos | action observation > scrambled control | 9 |
| 26 | Liew et al | 2011 | Familiarity modulates mirror neuron and mentalizing regions during intention understanding | 18 sub (mean age: 23.0) | videos of hand gestures, static controls | infer intentions | action observation > static control | 10 |
| 27 | Marsh et al | 2011 | Dissociation of mirroring and mentalising systems in autism | 19 sub (mean age: 32.2) | videos of grasping actions, moving shape controls | passive viewing | action observation > abstract control | 11 |
| 28 | Mazzarella et al | 2013 | Brain systems for visual perspective taking and action perception | 20 sub (mean age: 27.7) | pictures of reaching actions, background-only controls | visual perspective taking | action observation > background control | 16 |
| 29 | Mazzola et al | 2013 | Effects of emotional contexts on cerebello-thalamo-cortical activity during action observation | 23 sub (mean age: 29) | videos of grasping actions, videos of faces | passive viewing | action observation > face observation | 6 |
| 30 | Molinari et al | 2013 | Human parietofrontal networks related to action observation detected at rest | 16 sub (mean age: 23.3) | videos of grasping actions, static controls | passive viewing | action observation > static control | 3 |
| 31 | Morales et al | 2019 | An fMRI study of action observation and action execution in childhood | 18 sub (mean age: 22.93) | videos of hand actions, static controls | passive viewing | action observation > static control | 20 |
| 32 | Pierno et al | 2009 | Neurofunctional modulation of brain regions by the observation of pointing and grasping actions | 15 sub (mean age: 34) | pictures of grasping/pointing actions, no action controls | passive viewing | action observation > no action control | 12 |
| 33 | Pierno et al | 2006 | When gaze turns into grasp | 14 sub (mean age: 28) | videos of reaching actions, no action controls | passive viewing | action observation > no action control | 9 |
| 34 | Pierno et al | 2006 | Transfer of interfered motor patterns to self from others | 14 sub (mean age: 28) | videos of grasping actions, no action controls | passive viewing | action observation > no action control | 13 |
| 35 | Pilgramm et al | 2010 | Differential activation of the lateral premotor cortex during action observation | 36 sub (mean age: 26.5) | videos of dance moves, scrambled controls | passive viewing | action observation > scrambled control | 9 |
| 36 | Plata-Bello et al | 2017 | The Mirror Neuron System in Relapsing Remitting Multiple Sclerosis Patients with Low Disability | 15 sub (mean age: 44.2) | videos of hand actions, static controls | passive viewing | action observation > static control | 3 |
| 37 | Plata-Bello et al | 2014 | The mirror neuron system and motor dexterity: what happens? | 31 sub (mean age: 26.1) | videos of hand actions, static controls | passive viewing | action observation > static control | 14 |
| 38 | Plata-Bello et al | 2013 | Observation of simple intransitive actions: the effect of familiarity | 19 sub (mean age: 22.7) | videos of finger movements, static controls | passive viewing | action observation > static control | 35 |
| 39 | Schubotz et al | 2004 | Sequences of abstract nonbiological stimuli share ventral premotor cortex with action observation and imagery | 18 sub (mean age: 25.9) | videos of hand actions, abstract controls | detect errors/judge object properties | action observation > abstract control | 32 |
| 40 | Shmuelof & Zohary | 2006 | A mirror representation of others' actions in the human anterior parietal cortex | 14 sub (age range: 25-35) | videos of grasping actions, scrambled controls | passive viewing | action observation > scrambled control | 15 |
| 41 | Sokolov et al | 2012 | Biological motion processing: the left cerebellum communicates with the right superior temporal sulcus | 13 sub (mean age: 28.2) | videos of point-light walking, scrambled controls | detect repeated stimuli | action observation > scrambled control | 9 |
| 42 | Thakkar et al | 2014 | Altered brain activation during action imitation and observation in schizophrenia: a translational approach to investigating social dysfunction in schizophrenia | 16 sub (mean age: 37.4) | videos of button presses, static controls | passive viewing | action observation > static control | 2 |
| 43 | Thompson et al | 2007 | Common and distinct brain activation to viewing dynamic sequences of face and hand movements | 11 sub (age range: 22-40) | 3D animations of face/hand movements, abstract controls | detect stimuli sequences | action observation > abstract control | 8 |
| 44 | Tubaldi et al | 2011 | Smelling odors, understanding actions | 15 sub (mean age: 26) | videos of grasping actions, static controls | passive viewing | action observation > static control | 10 |
| 45 | Turella et al | 2011 | Object presence modulates activity within the somatosensory component of the action observation network | 19 sub (mean age: 27.2) | videos of grasping actions, static controls | passive viewing | action observation > static control | 17 |
| 46 | Turella et al | 2009 | Visual features of an observed agent do not modulate human brain activity during action observation | 17 sub (mean age: 27.8) | videos of grasping actions, static controls | passive viewing | action observation > static control | 16 |
| 47 | Villiger et al | 2013 | Enhanced activation of motor execution networks using action observation combined with imagination of lower limb movements | 14 sub (mean age: 25) | videos of kicking actions, scrambled controls | passive viewing | action observation > scrambled control | 7 |
| 48 | Vingerhoets et al | 2012 | Influence of perspective on the neural correlates of motor resonance during natural action observation | 17 sub (mean age: 21.8) | videos of grasping actions, object controls | discern type of grasp | action observation > object control | 38 |
| 49 | Wheaton et al | 2004 | Viewing the motion of human body parts activates different regions of premotor, temporal, and parietal cortex | 12 sub (mean age: 29.6) | videos of face/hand/leg actions, static controls | passive viewing | action observation > static control | 24 |
| 50 | Whitehead et al | 2009 | Neural correlates of observing pretend play in which one object is represented as another | 15 sub (mean age: 24.85) | videos of various actions, background controls | passive viewing | action observation > background control | 20 |
| 51 | Zhang et al | 2017 | Different activity patterns for action and language within their shared neural areas: An fMRI study on action observation and language phonology | 18 sub (mean age: 22.94) | pictures of hand actions, scrambled controls | detect duplicates/differences | action observation > landscape control | 6 |
|  |  |  |  | **TOT SUB: 933** |  |  |  | **TOT FOCI: 1006** |

**Supplementary Table 3**: Overview of the 51 studies included in the meta-analysis on the neural bases of action representational processing. N: progressive study number; Sub: subjects

| **N** | **Author** | **Year** | **Title** | **Sub (mean age)** | **Stimuli** | **Task** | **Contrast** | **Foci** |
| --- | --- | --- | --- | --- | --- | --- | --- | --- |
| 1 | Brass et al | 2001 | The inhibition of imitative response tendencies | 10 sub (mean age: 23.5) | videos of finger movements | perform specific finger movement | incongruent > congruent | 5 |
| 2 | Brass et al | 2005 | The inhibition of imitative and overlearned responses: a functional double dissociation | 20 sub (mean age: 26) | videos of hand actions | perform cued hand movement | incongruent > congruent | 7 |
| 3 | Campbell et al | 2018 | Intentionally not imitating: Insula cortex engaged for top-down control of action mirroring | 24 sub (mean age: 23.5) | videos of hand actions | perform specific action, copy/oppose | incongruent > congruent | 25 |
| 4 | Crescentini et al | 2011 | The effect of observed biological and non biological movements on action imitation: an fMRI study | 19 sub (mean age: 24.6) | videos of finger movements or moving dots | perform specific finger movement | incongruent > congruent | 1 |
| 5 | Cross et al | 2013 | Controlling automatic imitative tendencies: interactions between mirror neuron and cognitive control systems | 20 sub (age range: 19-39) | videos of finger movements or moving dots | perform specific finger movement | incongruent > congruent | 11 |
| 6 | Cross et al | 2013 | Optimized neural coding? Control mechanisms in large cortical networks implemented by connectivity changes | 24 sub (mean age: 22.4) | videos of finger movements | perform cued finger movement | incongruent > congruent | 24 |
| 7 | Darda et al | 2018 | Functional Specificity and Sex Differences in the Neural Circuits Supporting the Inhibition of Automatic Imitation | 28 sub (mean age: 23.96) | images of finger movements | perform cued finger movement | incongruent > congruent | 28 |
| 8 | Marsh et al | 2016 | The imitation game: Effects of social cues on ‘imitation’ are domain-general in nature | 24 sub (mean age: 23.71) | videos of finger movements with social cues | perform cued finger movement | incongruent > congruent | 30 |
| 9 | Mengotti et al | 2012 | Imitation components in the human brain: An fMRI study | 22 sub (mean age: 24.4) | videos of finger movements | spacial/anatomical imitation | non-specular > specular | 5 |
| 10 | Newman-Norlund et al | 2007 | The mirror neuron system is more active during complementary compared with imitative action | 19 sub (age range: 18-25) | sequences of object manipulations | manipulate object | incongruent > congruent | 5 |
| 11 | Sasaki et al | 2018 | Distinct sensitivities of the lateral prefrontal cortex and extrastriate body area to contingency between executed and observed actions | 24 sub (mean age: 24.8) | videos of finger movements | perform cued finger movement | incongruent > congruent | 3 |
| 12 | Spengler et al | 2009 | Control of shared representations relies on key processes involved in mental state attribution | 18 sub (mean age: 25) | videos of finger movements | perform cued finger movement | incongruent > congruent | 3 |
| 13 | Stanley et al | 2007 | Functional activation in parieto-premotor and visual areas dependent on congruency between hand movement and visual stimuli during motor-visual priming | 16 sub (age range: 18-48) | 3D animations of hand movements | perform specific hand movement | incongruent > congruent | 7 |
| 14 | Vrticka et al | 2013 | Neural substrates of social emotion regulation: a fMRI study on imitation and expressive suppression to dynamic facial signals | 20 sub (mean age: 33.5) | videos of facial emotional expressions | imitation or expressive suppression | expressive suppression > imitation | 2 |
| 15 | Wang et al | 2011 | The Control of Mimicry by Eye Contact Is Mediated by Medial Prefrontal Cortex | 20 sub (mean age: 23) | videos of hand and head actions | perform specific hand movement | incongruent > congruent | 38 |
|  |  |  |  | **TOT SUB: 308** |  |  |  | **TOT FOCI: 194** |

**Supplementary Table 4**: Overview of the 15 studies included in the meta-analysis on the neural bases of action relational processing. N: progressive study number; Sub: subjects

**References:**

Abdollahi, R. O., Jastorff, J., & Orban, G. A. (2012). Common and Segregated Processing of Observed Actions in Human SPL. *Cerebral Cortex*, *23*(11), 2734–2753. <https://doi.org/10.1093/cercor/bhs264>

Abraham, A., Rakoczy, H., Werning, M., von Cramon, D. Y., & Schubotz, R. I. (2010). Matching mind to world and vice versa: Functional dissociations between belief and desire mental state processing. *Social Neuroscience*, *5*(1), 1–18. <https://doi.org/10.1080/17470910903166853>

Abraham, A., Werning, M., Rakoczy, H., von Cramon, D. Y., & Schubotz, R. I. (2008). Minds, persons, and space: An fMRI investigation into the relational complexity of higher-order intentionality. *Consciousness and Cognition*, *17*(2), 438–450. <https://doi.org/10.1016/j.concog.2008.03.011>

Adamovich, S. V., August, K., Merians, A., & Tunik, E. (2009). A virtual reality-based system integrated with fmri to study neural mechanisms of action observation-execution: A proof of concept study. *Restorative Neurology and Neuroscience*, *27*(3), 209–223. <https://doi.org/10.3233/RNN-2009-0471>

Aichhorn, M., Perner, J., Weiss, B., Kronbichler, M., Staffen, W., & Ladurner, G. (2009). Temporo-parietal Junction Activity in Theory-of-Mind Tasks: Falseness, Beliefs, or Attention. *Journal of Cognitive Neuroscience*, *21*(6), 1179–1192. <https://doi.org/10.1162/jocn.2009.21082>

Alderson-Day, B., Weis, S., McCarthy-Jones, S., Moseley, P., Smailes, D., & Fernyhough, C. (2016). The brain’s conversation with itself: Neural substrates of dialogic inner speech. *Social Cognitive and Affective Neuroscience*, *11*(1), 110–120. <https://doi.org/10.1093/scan/nsv094>

Andrews-Hanna, J. R., Saxe, R., & Yarkoni, T. (2014). Contributions of episodic retrieval and mentalizing to autobiographical thought: Evidence from functional neuroimaging, resting-state connectivity, and fMRI meta-analyses. *NeuroImage*, *91*, 324–335. <https://doi.org/10.1016/j.neuroimage.2014.01.032>

Arioli, M., Perani, D., Cappa, S., Proverbio, A. M., Zani, A., Falini, A., & Canessa, N. (2018). Affective and cooperative social interactions modulate effective connectivity within and between the mirror and mentalizing systems. *Human Brain Mapping*, *39*(3), 1412–1427. <https://doi.org/10.1002/hbm.23930>

Bach, P., Peelen, M. V., & Tipper, S. P. (2010). On the Role of Object Information in Action Observation: An fMRI Study. *Cerebral Cortex*, *20*(12), 2798–2809. <https://doi.org/10.1093/cercor/bhq026>

Bardi, L., Desmet, C., Nijhof, A., Wiersema, J. R., & Brass, M. (2017). Brain activation for spontaneous and explicit false belief tasks overlaps: New fMRI evidence on belief processing and violation of expectation. *Social Cognitive and Affective Neuroscience*, *12*(3), 391–400. <https://doi.org/10.1093/scan/nsw143>

Baumgaertner, A., Buccino, G., Lange, R., McNamara, A., & Binkofski, F. (2007). Polymodal conceptual processing of human biological actions in the left inferior frontal lobe: Conceptual processing of biological hand actions. *European Journal of Neuroscience*, *25*(3), 881–889. <https://doi.org/10.1111/j.1460-9568.2007.05346.x>

Biagi, L., Cioni, G., Fogassi, L., Guzzetta, A., & Tosetti, M. (2010). Anterior intraparietal cortex codes complexity of observed hand movements. *Brain Research Bulletin*, *81*(4–5), 434–440. <https://doi.org/10.1016/j.brainresbull.2009.12.002>

Brass, M., Zysset, S., & von Cramon, D. Y. (2001). The inhibition of imitative response tendencies. *NeuroImage*, *14*(6), 1416–1423. <https://doi.org/10.1006/nimg.2001.0944>

Brass, Marcel, Derrfuss, J., & von Cramon, D. Y. (2005). The inhibition of imitative and overlearned responses: A functional double dissociation. *Neuropsychologia*, *43*(1), 89–98. <https://doi.org/10.1016/j.neuropsychologia.2004.06.018>

Buccino, G., Binkofski, F., Fink, G. R., Fadiga, L., Fogassi, L., Gallese, V., Seitz, R. J., Zilles, K., Rizzolatti, G., & Freund, H. J. (2001). Action observation activates premotor and parietal areas in a somatotopic manner: An fMRI study. *The European Journal of Neuroscience*, *13*(2), 400–404. <https://doi.org/10.1111/j.1460-9568.2001.01385.x>

Campbell, M. E. J., Mehrkanoon, S., & Cunnington, R. (2018). Intentionally not imitating: Insula cortex engaged for top-down control of action mirroring. *Neuropsychologia*, *111*, 241–251. <https://doi.org/10.1016/j.neuropsychologia.2018.01.037>

Cheng, Y., Meltzoff, A. N., & Decety, J. (2007). Motivation modulates the activity of the human mirror-neuron system. *Cerebral Cortex (New York, N.Y.: 1991)*, *17*(8), 1979–1986. <https://doi.org/10.1093/cercor/bhl107>

Cheung, H., Chen, L., Szeto, C.-Y., Feng, G., Lu, G., Zhang, Z., Zhu, Z., & Wang, S. (2012). False belief and verb non-factivity: A common neural basis? *International Journal of Psychophysiology: Official Journal of the International Organization of Psychophysiology*, *83*(3), 357–364. <https://doi.org/10.1016/j.ijpsycho.2011.12.002>

Contreras, J. M., Schirmer, J., Banaji, M. R., & Mitchell, J. P. (2013). Common brain regions with distinct patterns of neural responses during mentalizing about groups and individuals. *Journal of Cognitive Neuroscience*, *25*(9), 1406–1417. <https://doi.org/10.1162/jocn_a_00403>

Corbo, D., & Orban, G. A. (2017). Observing Others Speak or Sing Activates Spt and Neighboring Parietal Cortex. *Journal of Cognitive Neuroscience*, *29*(6), 1002–1021. <https://doi.org/10.1162/jocn_a_01103>

Corradi-Dell’Acqua, C., Hofstetter, C., & Vuilleumier, P. (2014). Cognitive and affective theory of mind share the same local patterns of activity in posterior temporal but not medial prefrontal cortex. *Social Cognitive and Affective Neuroscience*, *9*(8), 1175–1184. <https://doi.org/10.1093/scan/nst097>

Cracco, E., Hudson, A. R., Van Hamme, C., Maeyens, L., Brass, M., & Mueller, S. C. (2020). Early interpersonal trauma reduces temporoparietal junction activity during spontaneous mentalising. *Social Cognitive and Affective Neuroscience*, *15*(1), 12–22. <https://doi.org/10.1093/scan/nsaa015>

Crescentini, C., Mengotti, P., Grecucci, A., & Rumiati, R. I. (2011). The effect of observed biological and non biological movements on action imitation: An fMRI study. *Brain Research*, *1420*, 80–92. <https://doi.org/10.1016/j.brainres.2011.08.077>

Cross, K. A., & Iacoboni, M. (2013). Optimized neural coding? Control mechanisms in large cortical networks implemented by connectivity changes. *Human Brain Mapping*, *34*(1), 213–225. <https://doi.org/10.1002/hbm.21428>

Cross, K. A., Torrisi, S., Reynolds Losin, E. A., & Iacoboni, M. (2013). Controlling automatic imitative tendencies: Interactions between mirror neuron and cognitive control systems. *NeuroImage*, *83*, 493–504. <https://doi.org/10.1016/j.neuroimage.2013.06.060>

Darda, K. M., Butler, E. E., & Ramsey, R. (2018). Functional Specificity and Sex Differences in the Neural Circuits Supporting the Inhibition of Automatic Imitation. *Journal of Cognitive Neuroscience*, *30*(6), 914–933. <https://doi.org/10.1162/jocn_a_01261>

De Coster, L., Lin, L., Mathalon, D. H., & Woolley, J. D. (2019). Neural and behavioral effects of oxytocin administration during theory of mind in schizophrenia and controls: A randomized control trial. *Neuropsychopharmacology: Official Publication of the American College of Neuropsychopharmacology*, *44*(11), 1925–1931. <https://doi.org/10.1038/s41386-019-0417-5>

de Vega, M., León, I., Hernández, J. A., Valdés, M., Padrón, I., & Ferstl, E. C. (2014). Action sentences activate sensory motor regions in the brain independently of their status of reality. *Journal of Cognitive Neuroscience*, *26*(7), 1363–1376. <https://doi.org/10.1162/jocn_a_00559>

Di Cesare, G., Di Dio, C., Rochat, M. J., Sinigaglia, C., Bruschweiler-Stern, N., Stern, D. N., & Rizzolatti, G. (2014). The neural correlates of “vitality form” recognition: An fMRI study: this work is dedicated to Daniel Stern, whose immeasurable contribution to science has inspired our research. *Social Cognitive and Affective Neuroscience*, *9*(7), 951–960. <https://doi.org/10.1093/scan/nst068>

Di Dio, C., Di Cesare, G., Higuchi, S., Roberts, N., Vogt, S., & Rizzolatti, G. (2013). The neural correlates of velocity processing during the observation of a biological effector in the parietal and premotor cortex. *NeuroImage*, *64*, 425–436. <https://doi.org/10.1016/j.neuroimage.2012.09.026>

Dodell-Feder, D., Koster-Hale, J., Bedny, M., & Saxe, R. (2011). FMRI item analysis in a theory of mind task. *NeuroImage*, *55*(2), 705–712. <https://doi.org/10.1016/j.neuroimage.2010.12.040>

Dodell-Feder, D., Tully, L. M., Lincoln, S. H., & Hooker, C. I. (2014). The neural basis of theory of mind and its relationship to social functioning and social anhedonia in individuals with schizophrenia. *NeuroImage. Clinical*, *4*, 154–163. <https://doi.org/10.1016/j.nicl.2013.11.006>

Döhnel, K., Schuwerk, T., Meinhardt, J., Sodian, B., Hajak, G., & Sommer, M. (2012). Functional activity of the right temporo-parietal junction and of the medial prefrontal cortex associated with true and false belief reasoning. *NeuroImage*, *60*(3), 1652–1661. <https://doi.org/10.1016/j.neuroimage.2012.01.073>

Döhnel, K., Schuwerk, T., Sodian, B., Hajak, G., Rupprecht, R., & Sommer, M. (2017). An fMRI study on the comparison of different types of false belief reasoning: False belief-based emotion and behavior attribution. *Social Neuroscience*, *12*(6), 730–742. <https://doi.org/10.1080/17470919.2016.1241823>

Engel, A., Burke, M., Fiehler, K., Bien, S., & Rosler, F. (2008). How moving objects become animated: The human mirror neuron system assimilates non-biological movement patterns. *Social Neuroscience*, *3*(3–4), 368–387. <https://doi.org/10.1080/17470910701612793>

Fadiga, L., Craighero, L., Destro, M. F., Finos, L., Cotillon-Williams, N., Smith, A. T., & Castiello, U. (2006). Language in shadow. *Social Neuroscience*, *1*(2), 77–89. <https://doi.org/10.1080/17470910600976430>

Ferri, S., Rizzolatti, G., & Orban, G. A. (2015). The organization of the posterior parietal cortex devoted to upper limb actions: An fMRI study. *Human Brain Mapping*, *36*(10), 3845–3866. <https://doi.org/10.1002/hbm.22882>

Filimon, F., Nelson, J. D., Hagler, D. J., & Sereno, M. I. (2007). Human cortical representations for reaching: Mirror neurons for execution, observation, and imagery. *NeuroImage*, *37*(4), 1315–1328. <https://doi.org/10.1016/j.neuroimage.2007.06.008>

Georgescu, A. L., Kuzmanovic, B., Santos, N. S., Tepest, R., Bente, G., Tittgemeyer, M., & Vogeley, K. (2014). Perceiving nonverbal behavior: Neural correlates of processing movement fluency and contingency in dyadic interactions. *Human Brain Mapping*, *35*(4), 1362–1378. <https://doi.org/10.1002/hbm.22259>

Grèzes, J., Frith, C. D., & Passingham, R. E. (2004). Inferring false beliefs from the actions of oneself and others: An fMRI study. *NeuroImage*, *21*(2), 744–750. <https://doi.org/10.1016/S1053-8119(03)00665-7>

Grèzes, J., Pichon, S., & de Gelder, B. (2007). Perceiving fear in dynamic body expressions. *NeuroImage*, *35*(2), 959–967. <https://doi.org/10.1016/j.neuroimage.2006.11.030>

Grosbras, M.-H., & Paus, T. (2006). Brain Networks Involved in Viewing Angry Hands or Faces. *Cerebral Cortex*, *16*(8), 1087–1096. <https://doi.org/10.1093/cercor/bhj050>

Gweon, H., Dodell-Feder, D., Bedny, M., & Saxe, R. (2012). Theory of mind performance in children correlates with functional specialization of a brain region for thinking about thoughts. *Child Development*, *83*(6), 1853–1868. <https://doi.org/10.1111/j.1467-8624.2012.01829.x>

Hamilton, A. F. de C., Wolpert, D. M., Frith, U., & Grafton, S. T. (2006). Where does your own action influence your perception of another person’s action in the brain? *NeuroImage*, *29*(2), 524–535. <https://doi.org/10.1016/j.neuroimage.2005.07.037>

Hamzei, F., Rijntjes, M., Dettmers, C., Glauche, V., Weiller, C., & Büchel, C. (2003). The human action recognition system and its relationship to Broca’s area: An fMRI study. *NeuroImage*, *19*(3), 637–644. <https://doi.org/10.1016/s1053-8119(03)00087-9>

Hartwright, C. E., Apperly, I. A., & Hansen, P. C. (2012). Multiple roles for executive control in belief-desire reasoning: Distinct neural networks are recruited for self perspective inhibition and complexity of reasoning. *NeuroImage*, *61*(4), 921–930. <https://doi.org/10.1016/j.neuroimage.2012.03.012>

Hartwright, C. E., Apperly, I. A., & Hansen, P. C. (2014). Representation, control, or reasoning? Distinct functions for theory of mind within the medial prefrontal cortex. *Journal of Cognitive Neuroscience*, *26*(4), 683–698. <https://doi.org/10.1162/jocn_a_00520>

Hartwright, C. E., Apperly, I. A., & Hansen, P. C. (2015). The special case of self-perspective inhibition in mental, but not non-mental, representation. *Neuropsychologia*, *67*, 183–192. <https://doi.org/10.1016/j.neuropsychologia.2014.12.015>

Heitger, M. H., Macé, M. J.-M., Jastorff, J., Swinnen, S. P., & Orban, G. A. (2012). Cortical regions involved in the observation of bimanual actions. *Journal of Neurophysiology*, *108*(9), 2594–2611. <https://doi.org/10.1152/jn.00408.2012>

Hooker, C. I., Verosky, S. C., Germine, L. T., Knight, R. T., & D’Esposito, M. (2008). Mentalizing about emotion and its relationship to empathy. *Social Cognitive and Affective Neuroscience*, *3*(3), 204–217. <https://doi.org/10.1093/scan/nsn019>

Iacoboni, M., Molnar-Szakacs, I., Gallese, V., Buccino, G., Mazziotta, J. C., & Rizzolatti, G. (2005). Grasping the intentions of others with one’s own mirror neuron system. *PLoS Biology*, *3*(3), e79. <https://doi.org/10.1371/journal.pbio.0030079>

Iseki, K., Hanakawa, T., Shinozaki, J., Nankaku, M., & Fukuyama, H. (2008). Neural mechanisms involved in mental imagery and observation of gait. *NeuroImage*, *41*(3), 1021–1031. <https://doi.org/10.1016/j.neuroimage.2008.03.010>

Jacoby, N., Bruneau, E., Koster-Hale, J., & Saxe, R. (2016). Localizing Pain Matrix and Theory of Mind networks with both verbal and non-verbal stimuli. *NeuroImage*, *126*, 39–48. <https://doi.org/10.1016/j.neuroimage.2015.11.025>

Jastorff, J., Abdollahi, R. O., Fasano, F., & Orban, G. A. (2016). Seeing biological actions in 3D: An fMRI study. *Human Brain Mapping*, *37*(1), 203–219. <https://doi.org/10.1002/hbm.23020>

Jenkins, A. C., Dodell-Feder, D., Saxe, R., & Knobe, J. (2014). The neural bases of directed and spontaneous mental state attributions to group agents. *PloS One*, *9*(8), e105341. <https://doi.org/10.1371/journal.pone.0105341>

Jimura, K., Konishi, S., Asari, T., & Miyashita, Y. (2010). Temporal pole activity during understanding other persons’ mental states correlates with neuroticism trait. *Brain Research*, *1328*, 104–112. <https://doi.org/10.1016/j.brainres.2010.03.016>

Kandylaki, K. D., Nagels, A., Tune, S., Wiese, R., Bornkessel-Schlesewsky, I., & Kircher, T. (2015). Processing of false belief passages during natural story comprehension: An fMRI study. *Human Brain Mapping*, *36*(11), 4231–4246. <https://doi.org/10.1002/hbm.22907>

Kobayashi, C., Glover, G. H., & Temple, E. (2006). Cultural and linguistic influence on neural bases of “Theory of Mind”: An fMRI study with Japanese bilinguals. *Brain and Language*, *98*(2), 210–220. <https://doi.org/10.1016/j.bandl.2006.04.013>

Lee, J., Horan, W. P., Wynn, J. K., & Green, M. F. (2016). Neural Correlates of Belief and Emotion Attribution in Schizophrenia. *PloS One*, *11*(11), e0165546. <https://doi.org/10.1371/journal.pone.0165546>

Lee, J., Quintana, J., Nori, P., & Green, M. F. (2011). Theory of mind in schizophrenia: Exploring neural mechanisms of belief attribution. *Social Neuroscience*, *6*(5–6), 569–581. <https://doi.org/10.1080/17470919.2011.620774>

Liew, S.-L., Han, S., & Aziz-Zadeh, L. (2011). Familiarity modulates mirror neuron and mentalizing regions during intention understanding. *Human Brain Mapping*, *32*(11), 1986–1997. <https://doi.org/10.1002/hbm.21164>

Marsh, L. E., & Hamilton, A. F. de C. (2011). Dissociation of mirroring and mentalising systems in autism. *NeuroImage*, *56*(3), 1511–1519. <https://doi.org/10.1016/j.neuroimage.2011.02.003>

Marsh, L. E., Bird, G., & Catmur, C. (2016). The imitation game: Effects of social cues on “imitation” are domain-general in nature. *NeuroImage*, *139*, 368–375. <https://doi.org/10.1016/j.neuroimage.2016.06.050>

Mazzarella, E., Ramsey, R., Conson, M., & Hamilton, A. (2013). Brain systems for visual perspective taking and action perception. *Social Neuroscience*, *8*(3), 248–267. <https://doi.org/10.1080/17470919.2012.761160>

Mazzola, V., Vuilleumier, P., Latorre, V., Petito, A., Gallese, V., Popolizio, T., Arciero, G., & Bondolfi, G. (2013). Effects of emotional contexts on cerebello-thalamo-cortical activity during action observation. *PloS One*, *8*(9), e75912. <https://doi.org/10.1371/journal.pone.0075912>

Mengotti, P., Corradi-Dell’acqua, C., & Rumiati, R. I. (2012). Imitation components in the human brain: An fMRI study. *NeuroImage*, *59*(2), 1622–1630. <https://doi.org/10.1016/j.neuroimage.2011.09.004>

Mitchell, J. P. (2008). Activity in right temporo-parietal junction is not selective for theory-of-mind. Cereb Cortex, 18(2), 262-271. doi: 10.1093/cercor/bhm051

Molinari, E., Baraldi, P., Campanella, M., Duzzi, D., Nocetti, L., Pagnoni, G., & Porro, C. A. (2013). Human Parietofrontal Networks Related to Action Observation Detected at Rest. *Cerebral Cortex*, *23*(1), 178–186. <https://doi.org/10.1093/cercor/bhr393>

Morales, S., Bowman, L. C., Velnoskey, K. R., Fox, N. A., & Redcay, E. (2019). An fMRI study of action observation and action execution in childhood. *Developmental Cognitive Neuroscience*, *37*, 100655. <https://doi.org/10.1016/j.dcn.2019.100655>

Moran, J. M., Jolly, E., & Mitchell, J. P. (2012). Social-cognitive deficits in normal aging. *The Journal of Neuroscience: The Official Journal of the Society for Neuroscience*, *32*(16), 5553–5561. <https://doi.org/10.1523/JNEUROSCI.5511-11.2012>

Newman-Norlund, R. D., van Schie, H. T., van Zuijlen, A. M. J., & Bekkering, H. (2007). The mirror neuron system is more active during complementary compared with imitative action. *Nature Neuroscience*, *10*(7), 817–818. <https://doi.org/10.1038/nn1911>

Oliver, L. D., Vieira, J. B., Neufeld, R. W. J., Dziobek, I., & Mitchell, D. G. V. (2018). Greater involvement of action simulation mechanisms in emotional vs cognitive empathy. Soc Cogn Affect Neurosci, 13(4), 367-380. doi: 10.1093/scan/nsy013

Özdem, C., Brass, M., Schippers, A., Van der Cruyssen, L., & Van Overwalle, F. (2019). The neural representation of mental beliefs held by two agents. *Cognitive, Affective & Behavioral Neuroscience*, *19*(6), 1433–1443. <https://doi.org/10.3758/s13415-019-00714-2>

Özdem, C., Brass, M., Van der Cruyssen, L., & Van Overwalle, F. (2017). The overlap between false belief and spatial reorientation in the temporo-parietal junction: The role of input modality and task. *Social Neuroscience*, *12*(2), 207–217. <https://doi.org/10.1080/17470919.2016.1143027>

Pierno, A. C., Becchio, C., Wall, M. B., Smith, A. T., & Castiello, U. (2006). Transfer of interfered motor patterns to self from others. *The European Journal of Neuroscience*, *23*(7), 1949–1955. <https://doi.org/10.1111/j.1460-9568.2006.04706.x>

Pierno, A. C., Becchio, C., Wall, M. B., Smith, A. T., Turella, L., & Castiello, U. (2006). When gaze turns into grasp. *Journal of Cognitive Neuroscience*, *18*(12), 2130–2137. <https://doi.org/10.1162/jocn.2006.18.12.2130>

Pierno, A. C., Tubaldi, F., Turella, L., Grossi, P., Barachino, L., Gallo, P., & Castiello, U. (2009). Neurofunctional modulation of brain regions by the observation of pointing and grasping actions. *Cerebral Cortex (New York, N.Y.: 1991)*, *19*(2), 367–374. <https://doi.org/10.1093/cercor/bhn089>

Pilgramm, S., Lorey, B., Stark, R., Munzert, J., Vaitl, D., & Zentgraf, K. (2010). Differential activation of the lateral premotor cortex during action observation. *BMC Neuroscience*, *11*, 89. <https://doi.org/10.1186/1471-2202-11-89>

Plata Bello, J., Modroño, C., Marcano, F., & González–Mora, J. L. (2014). The mirror neuron system and motor dexterity: What happens? *Neuroscience*, *275*, 285–295. <https://doi.org/10.1016/j.neuroscience.2014.06.010>

Plata Bello, Julio, Modroño, C., Marcano, F., & González-Mora, J. L. (2013). Observation of simple intransitive actions: The effect of familiarity. *PloS One*, *8*(9), e74485. <https://doi.org/10.1371/journal.pone.0074485>

Plata-Bello, J., Pérez-Martín, Y., Castañón-Pérez, A., Modroño, C., Fariña, H., Hernández-Martín, E., González-Platas, M., Marcano, F., & González-Mora, J. L. (2017). The Mirror Neuron System in Relapsing Remitting Multiple Sclerosis Patients with Low Disability. *Brain Topography*, *30*(4), 548–559. <https://doi.org/10.1007/s10548-017-0558-y>

Rothmayr, C., Sodian, B., Hajak, G., Döhnel, K., Meinhardt, J., & Sommer, M. (2011). Common and distinct neural networks for false-belief reasoning and inhibitory control. *NeuroImage*, *56*(3), 1705–1713. <https://doi.org/10.1016/j.neuroimage.2010.12.052>

Sasaki, A. T., Okamoto, Y., Kochiyama, T., Kitada, R., & Sadato, N. (2018). Distinct sensitivities of the lateral prefrontal cortex and extrastriate body area to contingency between executed and observed actions. *Cortex; a Journal Devoted to the Study of the Nervous System and Behavior*, *108*, 234–251. <https://doi.org/10.1016/j.cortex.2018.08.003>

Saxe, R., & Powell, L. J. (2006). It’s the thought that counts: Specific brain regions for one component of theory of mind. *Psychological Science*, *17*(8), 692–699. <https://doi.org/10.1111/j.1467-9280.2006.01768.x>

Saxe, R., Schulz, L. E., & Jiang, Y. V. (2006). Reading minds versus following rules: dissociating theory of mind and executive control in the brain. Soc Neurosci, 1(3-4), 284-298. doi: 10.1080/17470910601000446

Schubotz, R. I., & von Cramon, D. Y. (2004). Sequences of abstract nonbiological stimuli share ventral premotor cortex with action observation and imagery. *The Journal of Neuroscience: The Official Journal of the Society for Neuroscience*, *24*(24), 5467–5474. <https://doi.org/10.1523/JNEUROSCI.1169-04.2004>

Schuwerk, T., Döhnel, K., Sodian, B., Keck, I. R., Rupprecht, R., & Sommer, M. (2014). Functional activity and effective connectivity of the posterior medial prefrontal cortex during processing of incongruent mental states. *Human Brain Mapping*, *35*(7), 2950–2965. <https://doi.org/10.1002/hbm.22377>

Shmuelof, L., & Zohary, E. (2006). A mirror representation of others’ actions in the human anterior parietal cortex. *The Journal of Neuroscience: The Official Journal of the Society for Neuroscience*, *26*(38), 9736–9742. <https://doi.org/10.1523/JNEUROSCI.1836-06.2006>

Sokolov, A. A., Erb, M., Gharabaghi, A., Grodd, W., Tatagiba, M. S., & Pavlova, M. A. (2012). Biological motion processing: The left cerebellum communicates with the right superior temporal sulcus. *NeuroImage*, *59*(3), 2824–2830. <https://doi.org/10.1016/j.neuroimage.2011.08.039>

Sommer, M., Döhnel, K., Jarvers, I., Blaas, L., Singer, M., Nöth, V., Schuwerk, T., & Rupprecht, R. (2018). False Belief Reasoning in Adults with and without Autistic Spectrum Disorder: Similarities and Differences. *Frontiers in Psychology*, *9*, 183. <https://doi.org/10.3389/fpsyg.2018.00183>

Sommer, M., Döhnel, K., Sodian, B., Meinhardt, J., Thoermer, C., & Hajak, G. (2007). Neural correlates of true and false belief reasoning. *NeuroImage*, *35*(3), 1378–1384. <https://doi.org/10.1016/j.neuroimage.2007.01.042>

Sommer, M., Meinhardt, J., Eichenmüller, K., Sodian, B., Döhnel, K., & Hajak, G. (2010). Modulation of the cortical false belief network during development. *Brain Research*, *1354*, 123–131. <https://doi.org/10.1016/j.brainres.2010.07.057>

Specht, K., & Wigglesworth, P. (2018). The functional and structural asymmetries of the superior temporal sulcus. *Scandinavian Journal of Psychology*, *59*(1), 74–82. <https://doi.org/10.1111/sjop.12410>

Spengler, S., von Cramon, D. Y., & Brass, M. (2009). Control of shared representations relies on key processes involved in mental state attribution. *Human Brain Mapping*, *30*(11), 3704–3718. <https://doi.org/10.1002/hbm.20800>

Stanley, J., & Miall, R. C. (2007). Functional activation in parieto-premotor and visual areas dependent on congruency between hand movement and visual stimuli during motor-visual priming. *NeuroImage*, *34*(1), 290–299. <https://doi.org/10.1016/j.neuroimage.2006.08.043>

Thakkar, K. N., Peterman, J. S., & Park, S. (2014). Altered brain activation during action imitation and observation in schizophrenia: A translational approach to investigating social dysfunction in schizophrenia. *The American Journal of Psychiatry*, *171*(5), 539–548. <https://doi.org/10.1176/appi.ajp.2013.13040498>

Thompson, J. C., Hardee, J. E., Panayiotou, A., Crewther, D., & Puce, A. (2007). Common and distinct brain activation to viewing dynamic sequences of face and hand movements. *NeuroImage*, *37*(3), 966–973. <https://doi.org/10.1016/j.neuroimage.2007.05.058>

Tubaldi, F., Turella, L., Pierno, A. C., Grodd, W., Tirindelli, R., & Castiello, U. (2011). Smelling odors, understanding actions. *Social Neuroscience*, *6*(1), 31–47. <https://doi.org/10.1080/17470911003691089>

Turella, L., Erb, M., Grodd, W., & Castiello, U. (2009). Visual features of an observed agent do not modulate human brain activity during action observation. *NeuroImage*, *46*(3), 844–853. <https://doi.org/10.1016/j.neuroimage.2009.03.002>

Turella, L., Tubaldi, F., Erb, M., Grodd, W., & Castiello, U. (2012). Object presence modulates activity within the somatosensory component of the action observation network. *Cerebral Cortex (New York, N.Y.: 1991)*, *22*(3), 668–679. <https://doi.org/10.1093/cercor/bhr140>

van der Meer, L., Groenewold, N. A., Nolen, W. A., Pijnenborg, M., & Aleman, A. (2011). Inhibit yourself and understand the other: Neural basis of distinct processes underlying Theory of Mind. *NeuroImage*, *56*(4), 2364–2374. <https://doi.org/10.1016/j.neuroimage.2011.03.053>

Villiger, M., Estévez, N., Hepp-Reymond, M.-C., Kiper, D., Kollias, S. S., Eng, K., & Hotz-Boendermaker, S. (2013). Enhanced activation of motor execution networks using action observation combined with imagination of lower limb movements. *PloS One*, *8*(8), e72403. <https://doi.org/10.1371/journal.pone.0072403>

Vingerhoets, G., Stevens, L., Meesdom, M., Honoré, P., Vandemaele, P., & Achten, E. (2012). Influence of perspective on the neural correlates of motor resonance during natural action observation. *Neuropsychological Rehabilitation*, *22*(5), 752–767. <https://doi.org/10.1080/09602011.2012.686885>

Völlm, B. A., Taylor, A. N. W., Richardson, P., Corcoran, R., Stirling, J., McKie, S., Deakin, J. F. W., & Elliott, R. (2006). Neuronal correlates of theory of mind and empathy: A functional magnetic resonance imaging study in a nonverbal task. *NeuroImage*, *29*(1), 90–98. <https://doi.org/10.1016/j.neuroimage.2005.07.022>

Vrticka, P., Simioni, S., Fornari, E., Schluep, M., Vuilleumier, P., & Sander, D. (2013). Neural substrates of social emotion regulation: A FMRI study on imitation and expressive suppression to dynamic facial signals. *Frontiers in Psychology*, *4*, 95. <https://doi.org/10.3389/fpsyg.2013.00095>

Wang, Y., Liu, W.-H., Li, Z., Wei, X.-H., Jiang, X.-Q., Neumann, D. L., Shum, D. H. K., Cheung, E. F. C., & Chan, R. C. K. (2015). Dimensional schizotypy and social cognition: An fMRI imaging study. *Frontiers in Behavioral Neuroscience*, *9*, 133. <https://doi.org/10.3389/fnbeh.2015.00133>

Wang, Y., Ramsey, R., & Hamilton, A. F. de C. (2011). The control of mimicry by eye contact is mediated by medial prefrontal cortex. *The Journal of Neuroscience: The Official Journal of the Society for Neuroscience*, *31*(33), 12001–12010. <https://doi.org/10.1523/JNEUROSCI.0845-11.2011>

Wheaton, K. J., Thompson, J. C., Syngeniotis, A., Abbott, D. F., & Puce, A. (2004). Viewing the motion of human body parts activates different regions of premotor, temporal, and parietal cortex. *NeuroImage*, *22*(1), 277–288. <https://doi.org/10.1016/j.neuroimage.2003.12.043>

Whitehead, C., Marchant, J. L., Craik, D., & Frith, C. D. (2009). Neural correlates of observing pretend play in which one object is represented as another. *Social Cognitive and Affective Neuroscience*, *4*(4), 369–378. <https://doi.org/10.1093/scan/nsp021>

Wysocka, J., Golec, K., Haman, M., Wolak, T., Kochański, B., & Pluta, A. (2020). Processing False Beliefs in Preschool Children and Adults: Developing a Set of Custom Tasks to Test the Theory of Mind in Neuroimaging and Behavioral Research. *Frontiers in Human Neuroscience*, *14*, 119. <https://doi.org/10.3389/fnhum.2020.00119>

Young, L., Dodell-Feder, D., & Saxe, R. (2010). What gets the attention of the temporo-parietal junction? An fMRI investigation of attention and theory of mind. *Neuropsychologia*, *48*(9), 2658–2664. <https://doi.org/10.1016/j.neuropsychologia.2010.05.012>

Zhang, Z., Sun, Y., Humphreys, G. W., & Song, Y. (2017). Different activity patterns for action and language within their shared neural areas: An fMRI study on action observation and language phonology. Neuropsychologia, 99, 112–120. HYPERLINK "https://doi.org/10.1016/j.neuropsychologia.2017.02.025"https://doi.org/10.1016/j.neuropsychologareas: An fMRI study on action observation and language phonology. *Neuropsychologia*, *99*, 112–120. <https://doi.org/10.1016/j.neuropsychologia.2017.02.025>
